# The role of C957T, TaqI and Ser311Cys polymorphisms of the DRD2 gene in schizophrenia: systematic review and meta-analysis

**DOI:** 10.1186/s12993-016-0114-z

**Published:** 2016-11-09

**Authors:** Thelma Beatriz González-Castro, Yazmín Hernández-Díaz, Isela Esther Juárez-Rojop, María Lilia López-Narváez, Carlos Alfonso Tovilla-Zárate, Alma Genis-Mendoza, Mariela Alpuin-Reyes

**Affiliations:** 1 División Académica Multidisciplinaria de Jalpa de Méndez, Universidad Juárez Autónoma de Tabasco, Jalpa de Méndez, Tabasco Mexico; 2 División Académica de Ciencias de la Salud, Universidad Juárez Autónoma de Tabasco, Villahermosa, Tabasco Mexico; 3 Secretaría de Salud, Hospital General de Yajalón, Yajalón, Chiapas Mexico; 4 División Académica Multidisciplinaria de Comalcalco, Universidad Juárez Autónoma de Tabasco, Ranchería Sur, Cuarta Sección, C.P. 86650 Comalcalco, Tabasco Mexico; 5 Secretaría de Salud, Instituto Nacional de Medicina Genómica (INMEGEN), Servicios de Atención Psiquiátrica (SAP), Ciudad de México, Mexico

**Keywords:** Schizophrenia, *DRD2* gene, Meta-analysis, Systematic review, Polymorphism

## Abstract

**Background:**

The association between the dopamine D2 receptor (*DRD2*) gene and schizophrenia has been studied though no conclusive outcomes have been attained. The aim of this study was to perform a systematic review and meta-analysis to explore the relation between three polymorphisms of the *DRD2* gene (C957T, TaqI and Ser311Cys) and schizophrenia.

**Methods:**

The search was made in PubMed and EBSCO databases (up to February 2016). The systematic review included 34 case–control association studies (34 for C957T, 16 for TaqI and 36 for Ser311Cys). The association analysis comprised the allelic, additive, dominant, and recessive genetic models. The meta-analysis was performed following the preferred reporting items for systematic reviews and meta-analyses (PRISMA) statement.

**Results:**

The meta-analysis showed that TaqI (additive model: OR 0.57, 95% CI 0.30–1.14) and C957T (additive model: OR 0.75, 95% OR 0.58–0.97, recessive model: OR 0.79, 95% CI 0.64–0.98) exert a protective effect against developing schizophrenia. However, the sub-analysis for the C957T variant showed that this polymorphism exhibits a risk factor effect on Chinese individuals (allelic model: OR 1.33, 95% CI 1.04–1.70).

**Conclusion:**

Our meta-analysis suggests an association of the *DRD2* gene and the risk for schizophrenia, given that TaqI and C957T polymorphisms presented a protective effect against schizophrenia, and in the sub-analyses the C957T variant increased the risk for this disorder in the Chinese population.

**Electronic supplementary material:**

The online version of this article (doi:10.1186/s12993-016-0114-z) contains supplementary material, which is available to authorized users.

## Background

Schizophrenia (SZ) is a common and complex multifactorial psychiatric disorder characterized by a variety of symptoms. These symptoms involve multiple psychological domains, including inferential thinking, attention, social interaction, expression of emotions, and volition. Typically, the onset of these symptoms starts manifesting in adolescence or early adulthood [1, 2]. Schizophrenia is a highly heritable and complex multifactorial illness; its heterogeneity is caused by both genetic and environmental factors and their interactions [3, 4]. High genetic risk for schizophrenia has led to considerable research efforts aimed at exploring its association with a number of candidate genes.

Although the biological etiology of schizophrenia is unknown, dopamine system dysfunction has been widely implicated in the pathogenesis of this disorder, and genes involved in dopaminergic pathways are being studied as candidate genes [5, 6]. Particular attention has been focused on the dopamine D2 receptor gene (*DRD2*). This is a transmembrane G protein-linked receptor which activates intracellular signaling by the inhibition of cAMP synthesis [7]. In humans, the *DRD2* gene is localized on chromosome 11 at the q22–q23 locus. This gene presents multiple polymorphisms, about 514 (http://snpper.chip.org/bio/snpper-enter/). From these, we selected three functional variants [8, 9]. The C957T (rs6277) variant constitutes a polymorphism with a synonymous coding C>T transition in exon 7. It has been proposed that this change influences the availability and affinity of the receptors [10–12]. Second, TaqI (rs1800497, C>T) comprises a substitution of an acidic amino acid for a basic one (Glu713Lys), and the two alleles are referred as A2 (cytosine) and A1 (thymine), respectively. The A1 allele is considered the risk allele [13, 14]. Finally, the Ser311Cys (rs1801028, C>G) polymorphism in exon 7 can present two variants, in which the C allele is the normal allele and encodes the amino acid serine (Ser) at codon 311, and the G allele is the risk allele and encodes a cysteine (Cys) [15, 16].

To date, a significant association between SZ and these functional *DRD2* gene polymorphisms (C957T, TaqI and Ser311Cys) has been reported by a number of authors [17–19]. However, several studies have failed to replicate this significant association [14, 20]. At least, two meta-analyses assessing the association between C957T, TaqI and Ser311Cys and schizophrenia have been performed. The first one was carried out by Yao et al. [21] in 2014 and the second by Li et al. [22] in 2015. Given that the dopamine system may contribute to the risk for schizophrenia, we conducted an update meta-analysis of all eligible published case–control studies to evaluate the effect of C957T, TaqI and Ser311Cys polymorphisms of the *DRD2* gene on the overall risk for SZ. The effects of ethnicity were also evaluated in this study.

## Methods

The search association between SZ and *DRD2* gene variants was performed according to the following assessments: (1) a meta-analysis of the TaqI polymorphism in subjects with SZ compared to healthy controls, (2) meta-analysis of the C957T polymorphism in subjects with SZ compared to healthy controls, (3) meta-analysis of the Ser311Cys polymorphism in subjects with SZ compared to healthy controls, (4) meta-analysis of the TaqI polymorphism in schizophrenics versus healthy controls in the Caucasian population, (5) meta-analysis of the C957T polymorphism in schizophrenics versus healthy controls in Caucasian and Asian populations, and a further analysis in Chinese and Japanese subjects, (6) meta-analysis of the Ser311Cys polymorphism in schizophrenics versus healthy controls by population. (7) Finally, a meta-regression method based on age including TaqI, C957T, and Ser311Cys polymorphisms was performed.

The meta-analyses were reported according to the preferred reporting items for systematic reviews and meta-analyses (PRISMA) statement [23, 24]. The PRISMA checklist is included as Additional file 1.

### Protocol registration

The protocol of this meta-analysis was registered in PROSPERO (http://www.crd.york.ac.uk/prospero/) with the registration number CRD42015029744.

### Publication search

To identify all potentially eligible studies on *DRD2* polymorphisms and schizophrenia risk, we performed a systematic search on PubMed and EBSCO databases that included all papers on the subject published up to February 2016. Relevant studies were identified using the terms: “*DRD2* AND C957T polymorphism AND schizophrenia”, “*DRD2* AND rs6277 AND schizophrenia”, “*DRD2* AND Ser311Cys polymorphism AND schizophrenia”, “*DRD2* AND rs1801028 AND schizophrenia”, “*DRD2* AND TaqI polymorphism AND schizophrenia”, “*DRD2* AND rs1800497 AND schizophrenia” “DRD AND rs6277”, “*DRD2* AND −141CInsDel”. References within the retrieved articles and review articles were also screened. Citation lists of retrieved articles were manually examined to ensure search sensitivity.

### Inclusion and exclusion criteria

Eligible studies had to meet the following criteria: (1) to be published in peer-reviewed journals, (2) to be designed as case–control studies, (3) to contain independent data, (4) to be association studies in which the frequencies of three genotypes were clearly stated or could be calculated, (5) inclusion of SZ diagnosis in the patient study group, and (6) the articles had to be written in English. Studies were excluded when: (1) they were not case–control studies, (2) they were reviews, comments or editorial articles, (3) they provided insufficient data, and (4) they were repeated studies.

### Data extraction

All the available data were extracted from each study by two researchers (Hernández-Díaz and González-Castro) working independently and in accordance with the inclusion criteria listed above. In case of disagreement in the inclusion, a third investigator was involved (Tovilla-Zárate) to resolve the discrepancy and a final decision was reached by the majority of votes. Data such as authors, year of publication, location, ethnic group, number of cases and controls, age, gender, SZ diagnosis of the participants and genotypes were collected.

### Publication bias

The possible presence of publication bias was evaluated graphically by drawing funnel plots and statistically by the Egger’s standard regression test. In the Egger’s test p < 0.10 was considered a statistically significant publication bias. The shape of the funnel plots serve as an indication of any obvious asymmetry for the TaqI, C957T and Ser311Cys variants, which was additionally supported by the Egger’s test. Moreover, to strengthen the analysis we evaluated publication bias by using the GRADE approach (Additional file 1). In addition, the 95% confidence interval (95% CI) of the effect size (ES) was also computed; effect size of 0.2 was regarded as small, effect size of 0.5 was considered moderate and ES greater than 0.8 was taken as large.

### Quality score assessment

For inclusion in the systematic review, each study was independently assessed by two reviewers (YHD and TBGC) using the Newcastle–Ottawa Assessment Scale (NOS) to estimate the methodological quality [25] (Table 1). The quality score of a given study was based on a score of six as cut-off point to distinguish high from low quality studies.

**Table 1:** Characteristics of the studies included in this meta-analysis

Author	Location	Nos	Number		Genotypes						p for HWE	
			Cases	Controls	Cases			Controls			Cases	Controls
					A1/A1	A1/A2	A2/A2	A1/A1	A1/A2	A2/A2		
Taq I												
Lafuente [43]	Spain	8	80	188	2	27	51	3	68	117	0.72	0.06
Monakhov [44]	Russia	8	311	364	189	104	18	238	116	10	0.51	0.48
Lafuente [45]	Spain	8	287	243	5	81	157	13	90	184	0.20	0.58
Behravan [17]	Iran	8	38	63	6	21	11	3	39	21	0.01^a^	0.52
Dubertret [46]	France	8	103	83	71	29	3	30	40	13	0.98	0.95
Aslan [14]	Turkey	8	99	109	2	97	0	0	106	3	0.00^a^	0.00^a^
Comings [63]	USA	4	87	69	58	27	2	59	10	0	0.56	0.37
Sanders [65]	USA	4	55	51	38	16	1	36	12	3	0.62	0.20
Campion [79]	France	5	80	80	60	19	1	58	20	2	0.70	0.86
Nöthen [56]	Germany	5	60	60	40	18	2	41	18	1	0.98	0.51
Dollfus [80]	France	6	62	61	41	19	2	11	45	5	0.91	0.00^a^
Jonsson [66]	Sweden	6	104	67	70	30	4	45	18	4	0.74	0.24
Dubertret [52]	France	7	50	50	36	13	1	26	21	3	0.88	0.63
Parsons [81]	Spain	8	119	165	92	24	3	93	68	4	0.39	0.04^a^
Vijayan [1]	India	8	212	194	102	93	17	88	77	29	0.62	0.08
Srivastava [61]	India	8	222	138	123	93	6	21	96	21	0.02^a^	0.00^a^

Author	Location	Nos	Number		Genotypes						p for HWE	
			Cases	Controls	Cases			Controls			Cases	Controls
					CC	CT17	TT	CC	107CT	TT		
C957T												
Jonsson [66]	Sweden	7	173	236	160	12	1	232	4	0	0.23	1.00
Lawford [11]	Australia	6	154	148	48	75	31	27	70	51	0.87	0.73
Hanninen [10]	Finland	7	188	384	59	92	37	104	176	104	0.91	0.102
Kukreti [47]	India	7	101	145	41	38	22	48	64	33	0.03^a^	0.23
Hoenicka [19]	Spain	7	131	364	30	61	40	46	174	144	0.48	0.65
Mo [48]	China	8	174	127	61	96	17	29	69	29	0.02^a^	0.37
Luo [49]	China	6	466	388	409	55	2	351	37	0	0.70	0.98
Monakhov [44]	Russia	8	311	364	99	152	60	78	183	103	0.90	0.91
Gupta [41]	India	8	254	225	104	112	38	76	120	29	0.41	0.09
Betcheva [12]	Bulgaria	8	255	556	58	128	66	192	253	111	0.89	0.09
Dubertret [46]	France	7	144	142	104	37	3	120	21	1	0.92	0.94
Fan [20]	China	8	421	403	366	52	3	368	34	1	0.43	0.55
Tsutsumi [42]	Japan	9	407	384	367	38	1	341	43	2	0.98	0.64
Arinami [27]	Japan	6	260	312	190	66	4	193	102	17	0.79	0.50
Li [82]	England	7	151	145	112	39	0	118	26	1	0.01^a^	0.72
Ohara [32]	Japan	7	170	121	136	34	0	84	36	1	0.37	0.30
Stöber [64]	Germany	7	260	290	207	50	3	236	53	1	0.99	0.21
Breen (1) [83]	England	7	378	292	293	78	7	227	61	4	0.47	0.96
Breen (2)	Scotland	7	151	145	115	33	3	118	26	1	0.71	0.72
Inada [84]	Japan	7	234	94	156	72	6	51	40	3	0.65	0.26
Tallerico [85]	Canada	7	50	51	40	10	0	43	7	1	0.29	0.36
Hori [39]	Japan	7	241	201	162	71	8	142	54	5	0.94	0.96
Himei [40]	Japan	7	190	103	118	69	3	71	27	5	0.06	0.30
Dubertret [52]	France	8	103	83	83	19	1	43	33	7	0.93	0.79
Kapman [86]	Finland	7	93	94	86	7	0	88	6	0	0.60	0.65
Parsons [81]	Spain	8	108	153	88	20	0	135	18	0	0.59	0.28
Lafuente [45]	Spain	8	243	291	208	33	2	235	54	2	0.63	0.75
Luu [67]	China	8	211	201	165	44	2	163	34	4	0.60	0.24
Sanders [57]	Europe	8	1870	2002	1495	354	21	1643	341	18	0.99	0.94
Cordeiro [68]	Brazil	8	229	733	183	38	8	498	206	29	0.00^a^	0.20
Srivastava [61]	India	8	233	224	161	65	7	172	48	4	0.81	0.75
Kurt [87]	Turkey	8	73	60	45	26	2	34	25	1	0.71	0.26
Saiz [88]	Spain	8	272	404	181	76	15	301	98	5	0.08	0.51
Xiao [69]	China	8	120	100	96	22	2	68	28	4	0.62	0.51

^a^Significant p value

### Statistical analysis

The comprehensive meta-analysis software (CMA, version 2) was used for the statistical analyses. The results are presented as odds ratios (ORs) and were used to assess the strength of the association between TaqI, C957T and Ser311Cys polymorphisms of the *DRD2* gene and SZ risk. Pooled ORs with their corresponding confidence intervals (95% CIs) were calculated for each of the models used: allelic (T vs C), additive (TT vs CC), dominant (TT + CT vs CC), and recessive (TT vs CT + TT). The estimated pooled ORs for each study were calculated using a random-effects model (Dersimonian and Laird method), though the fixed effects model was also considered (Mantel–Haenszel method). Heterogeneity of the studies was assessed with I^2^ and Q test statistics to identify significant outcomes. The sources of heterogeneity were also detected by sub-group analyses. Two sub-groups (Caucasian or Asian) according to different descents were analyzed for an ethnic-specific genetic comparison. Sample heterogeneity was analyzed with the Dersimonian and Laird’s Q test. Q test results were complemented with graphs to help the visualization of those studies favoring heterogeneity. The reliability of the results was assessed by sensitivity analysis performed for all outcomes to determine whether the results were driven mainly by single studies. In addition, we performed a meta-regression method based on age, to reduce the small sample size problem. We also performed a cumulative meta-analysis to provide a framework for updating the genetic effect of all studies. For the cumulative meta-analysis, studies were sorted chronologically by year of publication. The Hardy–Weinberg equilibrium (HWE) was checked using a Chi square test in each case and control group of the included studies; values of p < 0.05 were considered as showing a significant deviation from HWE. Finally, the strength of agreement between reviewers regarding study selection was evaluated by Kappa statistic.

## Results

### Characteristics of included studies

On-line literature search supplemented with a manual search resulted in 285 reports comprising 86 case–control studies [1, 10–12, 14–20, 22, 26–69], which were included in this meta-analysis (Table 1); this consisted of 18,692 SZ cases and 22,032 healthy controls. Of the 86 studies, 34 detailed the role of C957T in SZ, 36 examined the association of Ser311Cys with this disorder, and only 16 were available for the meta-analysis approach concerning the TaqI polymorphism and schizophrenia. In the case of TaqI, 12 studies were conducted in Caucasian populations, 2 in Indian, 1 in Iranian and 1 in Turkish populations, with a total of 1969 SZ cases and 1985 healthy controls. With regard to the C957T, 18 studies were conducted in Caucasians, 11 in Asians, 3 studies in Indians, 1 in Brazil and 1 in Turkish populations; in total 8819 SZ cases and 9965 healthy controls were included. Finally, for the Ser311Cys polymorphism, 18 studies were conducted in Asians, 15 in Caucasians and 3 in an Indian population with a total of 7827 SZ cases and 10,014 healthy controls. Characteristics of the 86 studies and the results of the HWE test are shown in Table 1.

### TaqI polymorphism and SZ

#### All populations

**Fig. 1:**
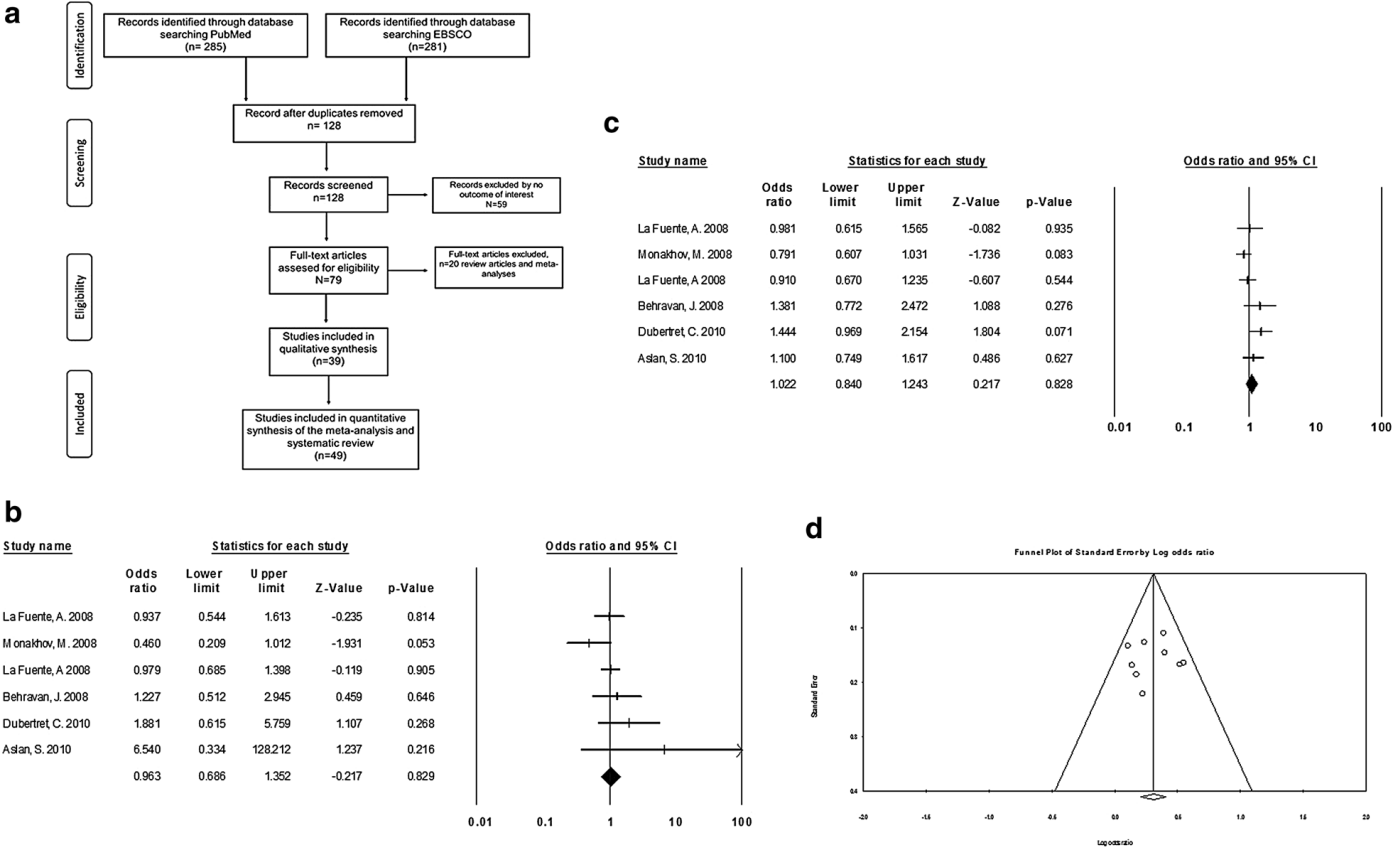
**a** Flow-chart design to show the inclusion of studies in this meta-analysis. **b** Forest plots of the allelic model for TaqI. **c** Forest plots of the dominant model for TaqI. **d** Begg’s funnel plot analysis of publication bias in the allelic model for TaqI

**Fig. 2:**
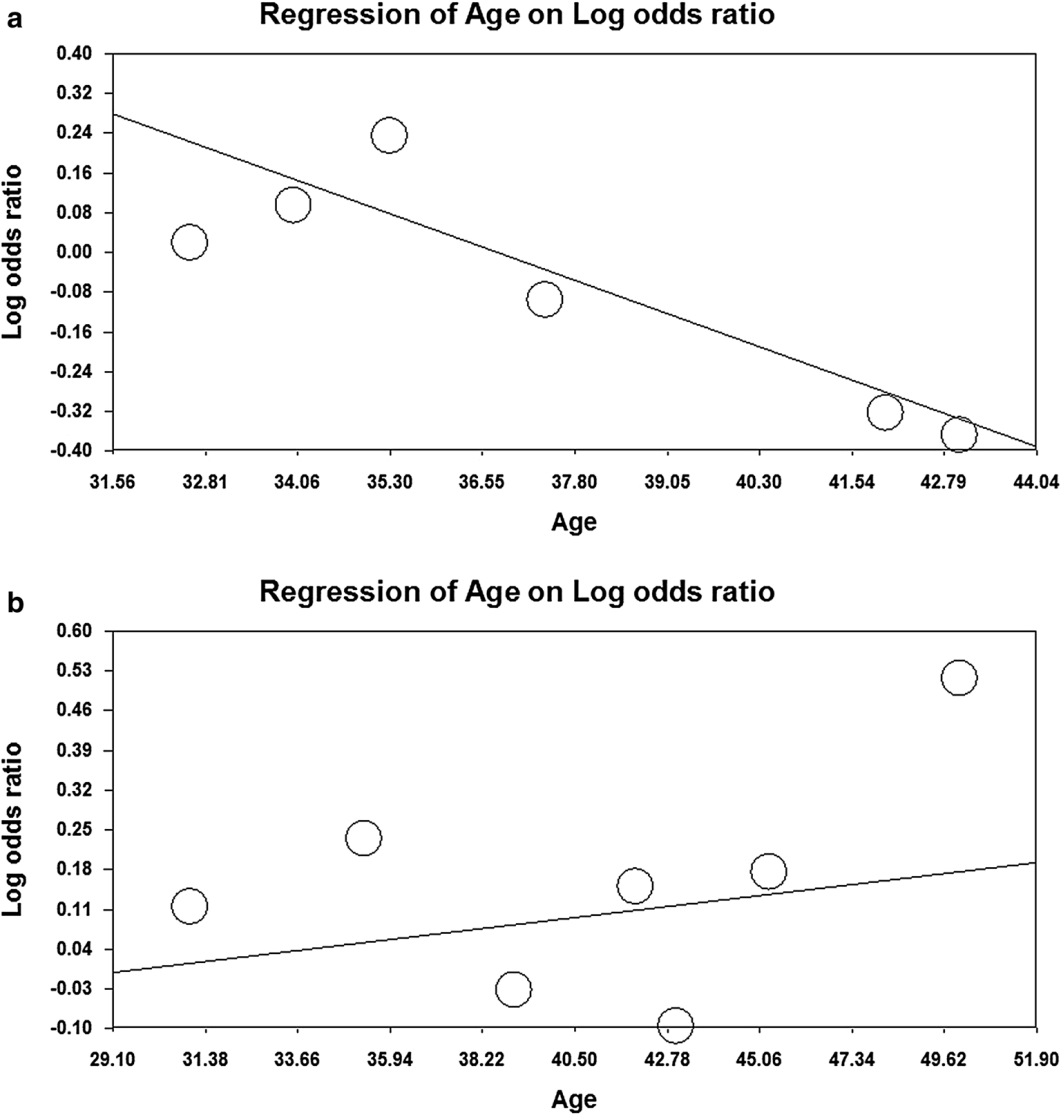
Meta-regression method based on age in the population as a whole. **a** TaqI polymorphism and **b** C957T polymorphism

Seventeen studies were included to identify the association between TaqI and SZ risk. Following the same pattern of analysis previously established for *DRD2* gene variants, all the genetic models: *allelic* (OR 0.92, 95% CI 0.71–1.19), *additive* (OR 0.59, 95% CI 0.30–1.14), *recessive* (OR 1.34, 95% CI 0.88–2.05) and *dominant* (OR 0.72, 95% CI 0.49–1.06) showed heterogeneity with p < 0.05. Subsequently, when we excluded the studies that favored the presence of the heterogeneity, we then observed the effect of the TaqI polymorphism in all populations using the *additive* genetic model (OR 0.57, 95% CI 0.38–0.86; p value of Q test: 0.32) and found a protective effect in the population as a whole. However, when we analyzed the *recessive model,* a risk effect was encountered (OR 1.50, 95% CI 1.10–2.03; p value of Q test: 0.66); see Table 2). The Egger’s test did not yield evidence of publication bias (Fig. 1). To reduce the effect of the small size of the sample in the analyses, we performed a meta-regression method based on age for the whole population. This analysis revealed a point estimate slope of −0.05365 and p value of 0.01686 (Fig. 2).

**Table 2:** Analysis of the association studies between the *DRD2* gene TaqI polymorphism and SZ in all populations and in a Caucasian sub-group

Model analysis		Model effects		p value of Q test
		Random OR (95% CI)	Fixed OR (95% CI)	
All populations				
Allelic	With heterogeneity	0.92 (0.71–1.19)	*0.89 (0.80–0.99)*	<0.00
	Without heterogeneity	0.92 (0.79–1.07)	0.92 (0.81–1.05)	0.256
Additive	With heterogeneity	0.59 (0.30–1.14)	*0.51 (0.37–0.71)*	<0.00
	Without heterogeneity	*0.57 (0.38–0.86)*	*0.57 (0.39–0.81)*	0.326
Recessive	With heterogeneity	1.34 (0.88–2.05)	1.17 (0.95–1.44)	<0.00
	Without heterogeneity	*1.50 (1.10–2.03)*	*1.50 (1.10–2.03)*	0.664
Dominant	With heterogeneity	0.72 (0.49–1.06)	*0.72 (0.62–0.84)*	<0.00
	Without heterogeneity	0.85 (0.72–1.01)	0.85 (0.72–1.01)	0.586
Caucasian population				
Allelic	With heterogeneity	0.88 (0.66–1.18)	*0.86 (0.77–0.96)*	<0.00
	Without heterogeneity	0.86 (0.71–1.05)	0.86 (0.71–1.05)	0.551
Additive	With heterogeneity			
	Without heterogeneity	*0.60 (0.36–0.99)*	*0.59 (0.39–0.91)*	0.263
Recessive	With heterogeneity			
	Without heterogeneity	0.90 (0.69–1.18)	0.90 (0.71–1.15)	0.403
Dominant	With heterogeneity	0.76 (0.50–1.14)	*0.77 (0.64–0.93)*	<0.00
	Without heterogeneity	0.89 (0.71–1.11)	0.89 (0.72–1.10)	0.397

Italic values denote significant value, p < 0.05

#### Caucasian population

**Fig. 3:**
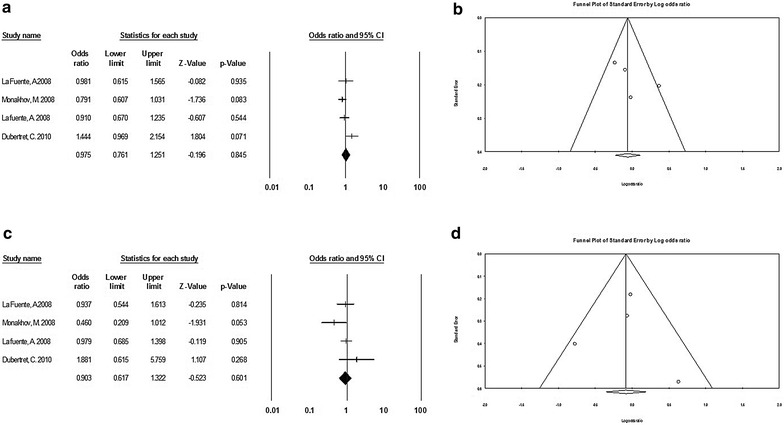
**a** Forest plots of the allelic model for TaqI in Caucasians. **b** Begg’s funnel plot analysis of publication bias of the allelic model for TaqI in Caucasians. **c** Forest plots of the dominant model for TaqI in Caucasians. **d** Begg’s funnel plot analysis of publication bias in the dominant model for TaqI in Caucasians

Given that previous studies have reported a positive association between TaqI and SZ risk in Caucasians [46], we decided to conduct a meta-analysis on the Caucasian population. This sub-group analysis by ethnicity included seven studies which showed no evidence of any association between TaqI and SZ in Caucasian populations. The results for the different genetic models were: *allelic* (OR 0.86, 95% CI 0.71–1.05; p value of Q test: 0.55), *recessive* (OR 0.90, 95% CI 0.69–1.18; p value of Q test: 0.40) and *dominant* (OR 0.89, 95% CI 0.71–1.11; p value of Q test: 0.39). However, in the *additive* model we observed a protective effect of TaqI on schizophrenia (OR 0.60, 95% CI 0.36–0.99; p value of Q test: 0.26) (Fig. 3).

### C957T polymorphism and schizophrenia

#### All populations

**Fig. 4:**
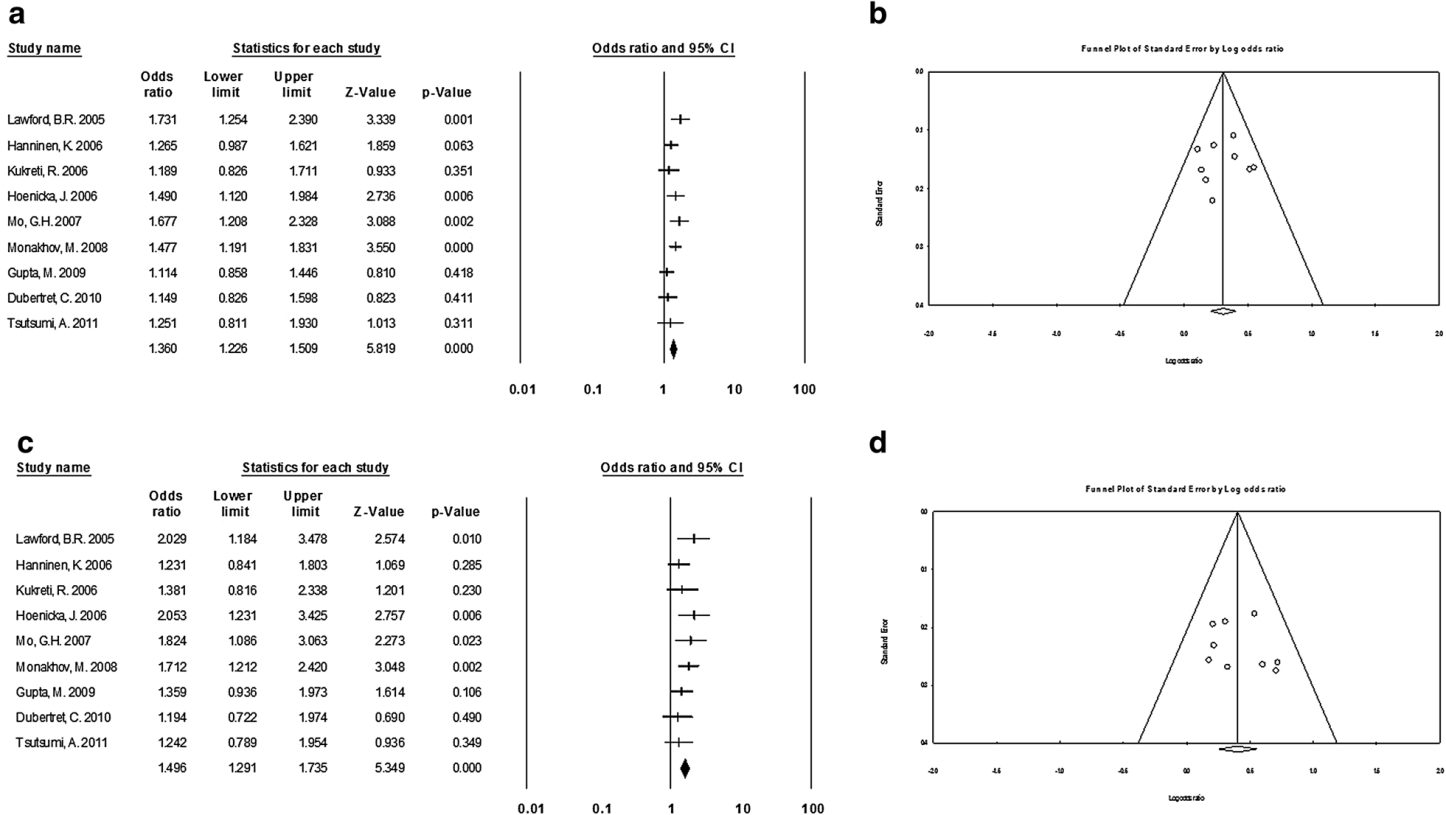
**a** Forest plots of the allelic model for C957T. **b** Begg’s funnel plot analysis of publication bias in the allelic model for C957T. **c** Forest plots of the dominant model for C957T. **d** Begg’s funnel plot analysis of publication bias in the dominant model for C957T

We performed an analysis in the population as a whole to explore the probable risk role of the C957T polymorphism in schizophrenia. Initially, we conducted a meta-analysis with the four genetic models proposed: *allelic* (OR 0.92, 95% CI 0.81–1.05), *additive* (OR 0.77, 95% CI 0.57–1.05), *recessive* (OR 0.84, 95% CI 0.66–1.06) and *dominant* (OR 0.91, 95% CI 0.78–1.05), in which p of Q test <0.05 indicated heterogeneity. No statistical association was found between the C957T polymorphism and schizophrenia. However, when we discarded the studies favoring heterogeneity, we obtained the following outcomes of statistical association for the models: *additive* (OR 0.75, 95% CI 0.58–0.97; p value of Q test: 0.15) and *recessive* (OR 0.79; 95% CI 0.64–0.98; p value of Q test: 0.21) (Table 3). In addition, the Egger’s test revealed no evidence of publication bias (Fig. 4). With regard to the meta-regression based on age, the slope was 0.00849 and the p value 0.38756 (Fig. 2).

**Table 3:** Analysis of association studies between the *DRD2* gene C957T polymorphism and schizophrenia by populations

Model analysis		Model effects		p value of Q test
		Random OR (95% CI)	Fixed OR (95% CI)	
All populations				
Allelic	With heterogeneity	0.92 (0.81–1.05)	*0.93 (0.87–0.98)*	<0.00
	Without heterogeneity	1.03 (0.93–1.15)	1.03 (0.93–1.15)	0.595
Additive	With heterogeneity	0.77 (0.57–1.05)	*0.76 (0.65–0.89)*	<0.00
	Without heterogeneity	*0.75 (0.58–0.97)*	*0.74 (0.61–0.91)*	0.151
Recessive	With heterogeneity	0.84 (0.66–1.06)	0.82 (0.72–0.94)	<0.00
	Without heterogeneity	*0.79 (0.64–0.98)*	*0.78 (0.66–0.92)*	0.211
Dominant	With heterogeneity	0.91 (0.78–1.05)	0.94 (0.88–1.01)	<0.00
	Without heterogeneity	0.89 (0.77–1.03)	0.89 (0.78–1.02)	0.308
Caucasian population				
Allelic	With heterogeneity	0.98 (0.81–1.18)	0.98 (0.91–1.05)	<0.00
	Without heterogeneity	1.03 (0.88–1.21)	1.00 (0.87–1.14)	0.252
Additive	With heterogeneity	0.85 (0.54–1.34)	*0.80 (0.66–0.97)*	<0.00
	Without heterogeneity	0.94 (0.63–1.40)	0.90 (0.63–1.27)	0.354
Recessive	With heterogeneity	0.89 (0.65–1.23)	*0.83 (0.71–0.98)*	<0.00
	Without heterogeneity	*0.73 (0.60–0.89)*	*0.73 (0.60–0.89)*	0.440
Dominant	With heterogeneity	0.98 (0.79–1.21)	1.03 (0.93–1.13)	<0.00
	Without heterogeneity	1.04 (0.89–1.21)	1.03 (0.89–1.20)	0.400
Asian population				
Allelic	With heterogeneity	0.84 (0.66–1.07)	*0.82 (0.73–0.93)*	<0.00
	Without heterogeneity	*0.66 (0.52–0.83)*	*0.66 (0.52–0.83)*	0.725
Additive	With heterogeneity			
	Without heterogeneity	*0.49 (0.28–0.86)*	*0.45 (0.29–0.70)*	0.206
Recessive	With heterogeneity			
	Without heterogeneity	*0.52 (0.32–0.83)*	*0.49 (0.32–0.75)*	0.330
Dominant	With heterogeneity	0.84 (0.64–1.10)	*0.85 (0.73–0.99)*	<0.00
	Without heterogeneity	*0.61 (0.50–0.74)*	*0.61 (0.50–0.74)*	0.864

Italic values denote significant value, p < 0.05

#### Caucasian population

**Fig. 5:**
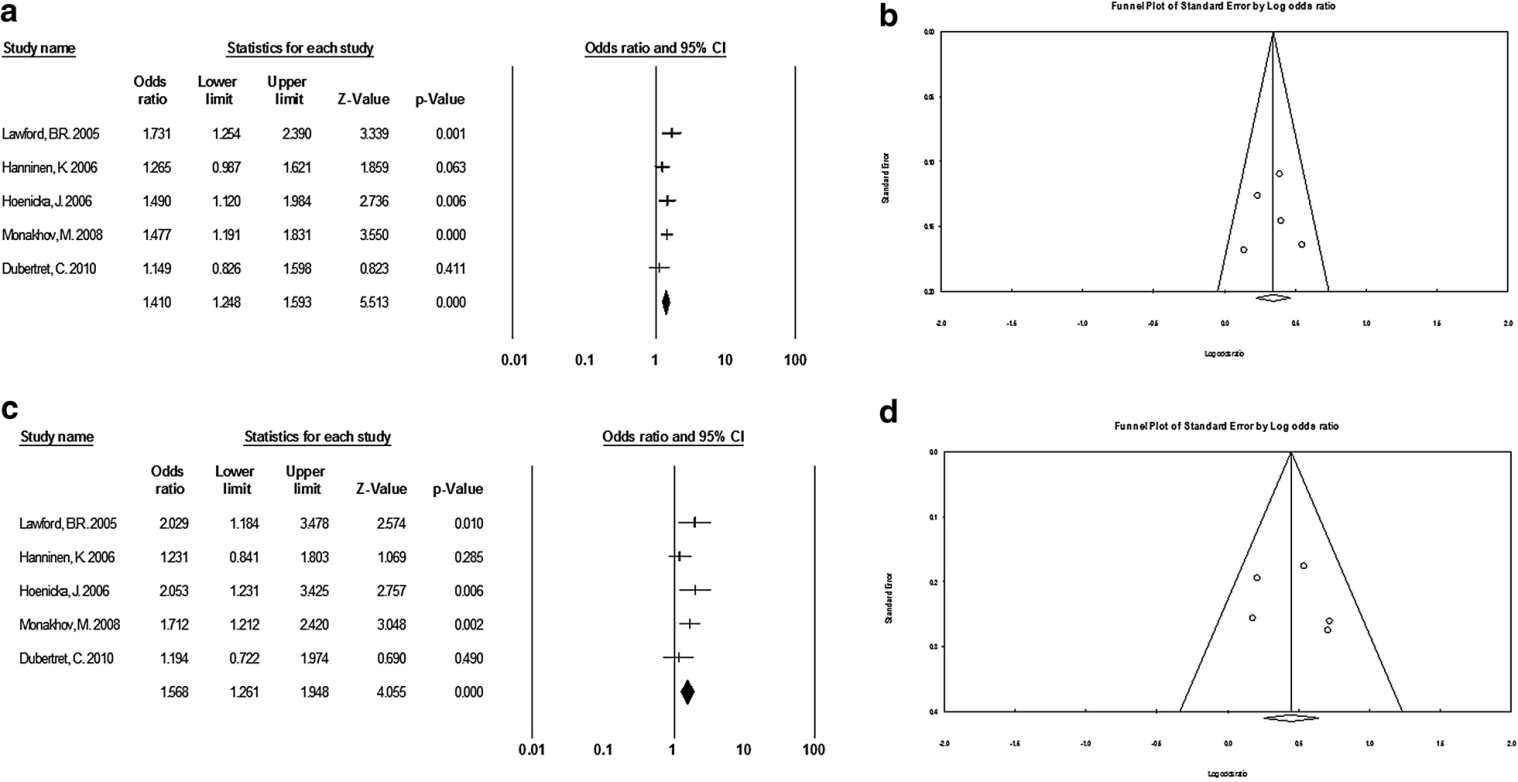
**a** Forest plots of the allelic model for C957T in Caucasians. **b** Begg’s funnel plot analysis of publication bias in the allelic model for C957T in Caucasians. **c** Forest plots of the dominant model for C957T in Caucasians. **d** Begg’s funnel plot analysis of publication bias in the dominant model for C957T in Caucasians

We performed a stratified analysis by ethnicity to measure SZ risk by populations. With regard to Caucasians, the first outcomes with a p of Q test <0.05 showed evidence of heterogeneity in the *allelic* (OR 0.98, 95% CI 0.81–1.18), *additive* (OR 0.85, 95% CI 0.54–1.34), *recessive* (OR 0.89, 95% CI 0.65–1.23) and *dominant* (OR 0.98, 95% CI 0.79–1.21) models. Subsequently, when heterogeneity was discarded, the outcome presented a positive association with schizophrenia in the *allelic* model (OR 0.73, 95% CI 0.60–0.89; p value of Q test: 0.44). However, a slight possibility of an association in the *additive* (OR 0.80, 95% CI 0.66–0.97; p value of Q test <0.00) and *recessive* (OR 0.83, 95% CI 0.71–0.98; p value of Q test <0.00) models could be suggested. But since these findings were in the presence of heterogeneity and using the fixed effects model, we did not consider them for the analysis. For all the analyses in Caucasians, the p value of the Egger’s test suggested the non-existence of publication bias (Fig. 5).

#### Asian population

Finally, for the C957T polymorphism in the Asian population we followed the same pattern of analysis as in the previous sub-section. In the initial analysis the outcomes exhibited the presence of heterogeneity (p < 0.05) in the *allelic* (OR 0.84, 95% CI 0.66–1.07) and *dominant* models (OR 0.84, 95% CI 0.64–1.10). After we excluded the studies that favored heterogeneity, the results evidenced an association between the C957T polymorphism and SZ in the four models: *allelic* (OR 0.66, 95% CI 0.52–0.83; p value of Q test: 0.72), *additive* (OR 0.49, 95% CI 0.28–0.86; p value of Q test: 0.20), *recessive* (OR 0.52, 95% CI 0.32–0.83; p value of Q test: 0.33) and *dominant* (OR 0.61, 95% CI 0.50–0.74; p value of Q test: 0.061), using the random effects method. However, we want to emphasize that the outcomes showed the same protective association between C957T and SZ in the all models when we used the fixed effects model.

### C957T polymorphism in Chinese and Japanese populations

In order to perform a more comprehensive and comparative meta-analysis we conducted two more sub-analyses, but only for the subjects born in Japan and in China. These sub-analyses helped to compare our findings with previous published met-analyses. Initially, we selected the studies that explored the role of C957T in Japanese schizophrenics and found a relation to SZ in the four models without heterogeneity, viz.: *allelic* (OR 0.69, 95% CI 0.57–0.85; p value of Q test: 0.11), *additive* (OR 0.51, 95% CI 0.27–0.95; p value of Q test: 0.24), *recessive* (OR 0.54, 95% CI 0.29–0.99; p value of Q test: 0.27) and *dominant* (OR 0.58, 95% CI 0.45–0.76; p value of Q test: 0.98), but all the results were for the fixed effects model. Nevertheless, when we used the random effects method we encountered the same pattern only in the *allelic* (OR 0.71, 95% CI 0.53–0.94) and *dominant* (OR 0.58, 95% CI 0.45–0.76) models. With regard to the Chinese population, we found a similar association to that of the previous sub-analysis. Interestingly, we found a risk effect in the *allelic* (OR 1.33, 95% CI 1.04–1.70; p value of Q test: 0.50) and *dominant* (OR 1.36, 95% CI 1.04–1.77; p value of Q test: 0.69) models, without heterogeneity using the random effects method (Table 4).

**Table 4:** Analysis of association studies between the *DRD2* C957T polymorphism and schizophrenia in China and Japan

Model analysis		Model effects		p value of Q test
		Random OR (95% CI)	Fixed OR (95% CI)	
Japan				
Allelic	With heterogeneity	0.79 (0.58–1.07)	*0.78 (0.66–0.93)*	0.017
	Without heterogeneity	*0.71 (0.53–0.94)*	*0.69 (0.57–0.85)*	0.112
Additive	With heterogeneity			
	Without heterogeneity	0.50 (0.24–1.07)	*0.51 (0.27–0.95)*	0.243
Recessive	With heterogeneity			
	Without heterogeneity	0.53 (0.26–1.08)	*0.54 (0.29–0.99)*	0.279
Dominant	With heterogeneity	0.79 (0.55–1.14)	*0.78 (0.64–0.96)*	0.011
	Without heterogeneity	*0.58 (0.45–0.76)*	*0.58 (0.45–0.76)*	0.988
China				
Allelic	With heterogeneity	0.95 (0.62–1.45)	0.92 (0.77–1.11)	<0.00
	Without heterogeneity	*1.33 (1.04–1.70)*	*1.33 (1.04–1.70)*	0.507
Additive	With heterogeneity			
	Without heterogeneity	0.55 (0.21–1.39)	*0.40 (0.22–0.73)*	0.173
Recessive	With heterogeneity			
	Without heterogeneity	0.54 (0.25–1.15)	*0.46 (0.26–0.79)*	0.283
Dominant	With heterogeneity	0.96 (0.62–1.48)	1.02 (0.82–1.28)	<0.00
	Without heterogeneity	*1.36 (1.04–1.77)*	*1.36 (1.04–1.77)*	0.697

Italic values denote significant value, p < 0.05

### Ser311Cys polymorphism and SZ

For this polymorphism the meta-analysis was performed for the overall population. The outcomes in Caucasian and Asian populations were similar to those found for the previous variants. Since the present work showed the same results as in previous studies [21], we will not discuss this polymorphism in the present work. However, we present the details in Additional file 2.

### Sensitivity analysis

In addition, a sensitivity analysis was carried out in which one study at a time was excluded to determine whether a specific study was favoring a marked heterogeneity. Nevertheless, the presence of heterogeneity was not explained by just one study. Furthermore, to measure the effects over time on the studies, we performed a cumulative meta-analysis, in which individual data sets were ordered chronologically (Additional file 3).

## Discussion

Schizophrenia is a complex genetic disorder manifesting combined environmental and genetic factors. Several studies have suggested that genetic variants of the *DRD2* gene play a role in SZ etiology [70, 71]. To assess the relationship between the *DRD2* genetic variants and the risk to develop schizophrenia, we conducted a meta-analysis of three *DRD2* polymorphisms: TaqI, C957T and Ser311Cys. The meta-analysis approach is a powerful tool to summarize contradicting results from different studies and has been used to analyze the role of various genes in schizophrenia [54, 72, 73].

First, we performed the analysis of the TaqI polymorphism to assess the role of this genetic variant in schizophrenia. There was a protective effect in the additive model in the population as a whole and in Caucasians. Also, we found a risk effect when using the recessive model in the combined results of the analysis for all populations. However, various studies have reported that TaqI polymorphism does not play an important role in the psychopathological symptoms of schizophrenia, whereas other researches agree with our results [21, 22, 63, 66]. One of the reasons for this discrepancy could be the relative small size of the sample, which limits the statistical power for the detection of a relationship between the TaqI polymorphism and schizophrenia [72]; more studies are needed to further validate these results. Another explanation is the environmental exposure that could trigger the expression of a gene, and this in turn could modify other genes which may then interact with *DRD2* and increase the risk to present the disease. In spite of the contrasting outcomes published, the role of TaqI has been more related to substance abuse, since the less frequent allele (A1 allele) has been associated with some psychiatric disorders such as alcoholism and substance abuse [74, 75]. On the other hand, previous studies have demonstrated that subjects with one or two A1 alleles of the *DRD2* polymorphism at the Taq1 A locus present lower *DRD2* density than those with no A1 allele [76]. Also, other studies have shown that female patients with the A1 allele exhibit greater prolactin response to nemonapride, a selective antagonist for D2-like dopamine receptors in schizophrenic patients [77]. Due to this association between TaqI and schizophrenia, the A1 allele has been suggested to diminish dopaminergic activity in the central nervous system [78].

For the C957T polymorphism, the comparisons performed in our study showed a significant positive association between this polymorphism and SZ in the overall population and in Caucasian and Asian sub-groups. In this sense, we recognize the existence of two previous meta-analyses [21, 22], in which many differences are observed: first, we identified a protective effect of the T allele of C957T using the additive and recessive models when analyzing the population as a whole, as well as when using the recessive model in Caucasians and the four genetic models in Asians. In contrast, Yao et al. did not observe any association. The differences could be due to the size of the samples. Our present study used 8819 SZ patients and 9965 healthy controls compared with 6075 SZ and 6643 controls of the previous meta-analysis by Yao et al. [21]. We included 2792 cases and 3322 controls more. In the Asian population a protective effect was found in all the models we used. As a consequence, we decided to perform an analysis by Asiatic subpopulations. Therefore, we divided the Asian population into Chinese and Japanese samples. In these sub-analyses we encountered unexpected results: the Chinese population showed an increased risk, whereas the Japanese population showed a protective association. This is clear an “allele paradox” between populations that may reflect the difference in the distribution of allele frequencies across the geographical localization. Our results draw attention to the influence of other factors such as the environment, which could be acting with ethnicity in this genetic association.

There are several limitations in this study. First, the sample size for some sub-group analyses was limited; therefore, more studies with larger samples should be included to enhance the reliability and stability of the meta-analysis. Second, a language bias may be present given that only studies published in English were included. Third, due to the limitation of the data, we did not stratify according to other potential factors which may enhance the risk for the development of SZ, such as gender, age of onset and clinical manifestations.

## Conclusions

The meta-analysis indicated that TaqI and C957T polymorphisms show a protective effect against SZ. In the sub-analysis of the C957T polymorphism we observed that this variant may contribute to the occurrence of schizophrenia in Chinese subjects, so the influence of ethnicity could be important in modifying the role of this polymorphism in SZ. Given the limitations of the studies included in the meta-analysis, future studies with larger samples and prospective designs are needed to fully understand the relationship between these polymorphisms and SZ. However, this meta-analysis still provides new insights into the role of the *DRD2* gene in SZ risk.

## Additional files

**Additional file 1.** Grades quality assessment and PRISMA check list.

**Additional file 2.** Association of the C957T polymorphism and schizophrenia.

**Additional file 3.** Sensitivity analysis and cumulative meta-analysis.

## Abbreviations

DRD2: dopamine D2 receptor
SZ: schizophrenia
NOS: Newcastle–Ottawa Assessment Scale
PRISMA: preferred reporting items for systematic reviews and meta-analyses
HWE: Hardy–Weinberg equilibrium

## References

[1] Vijayan NN, Bhaskaran S, Koshy LV, Natarajan C, Srinivas L, Nair CM, Allencherry PM, Banerjee M (2007). Association of dopamine receptor polymorphisms with schizophrenia and antipsychotic response in a South Indian population. Behav Brain Funct.

[2] Cannon TD (2015). How schizophrenia develops: cognitive and brain mechanisms underlying onset of psychosis. Trends Cogn Sci.

[3] Winchester CL, Pratt JA, Morris BJ (2014). Risk genes for schizophrenia: translational opportunities for drug discovery. Pharmacol Ther.

[4] Cannon TD, van Erp TG, Bearden CE, Loewy R, Thompson P, Toga AW, Huttunen MO, Keshavan MS, Seidman LJ, Tsuang MT (2003). Early and late neurodevelopmental influences in the prodrome to schizophrenia: contributions of genes, environment, and their interactions. Schizophr Bull.

[5] Moran PM, O’Tuathaigh CM, Papaleo F, Waddington JL (2014). Dopaminergic function in relation to genes associated with risk for schizophrenia: translational mutant mouse models. Prog Brain Res.

[6] Seeman P (2013). Schizophrenia and dopamine receptors. Eur Neuropsychopharmacol.

[7] Sumiyoshi T, Kunugi H, Nakagome K (2014). Serotonin and dopamine receptors in motivational and cognitive disturbances of schizophrenia. Front Neurosci.

[8] Noble EP (2000). The DRD2 gene in psychiatric and neurological disorders and its phenotypes. Pharmacogenomics.

[9] Hoenicka J, Aragues M, Ponce G, Rodriguez-Jimenez R, Jimenez-Arriero MA, Palomo T (2007). From dopaminergic genes to psychiatric disorders. Neurotox Res.

[10] Hanninen K, Katila H, Kampman O, Anttila S, Illi A, Rontu R, Mattila KM, Hietala J, Hurme M, Leinonen E (2006). Association between the C957T polymorphism of the dopamine D2 receptor gene and schizophrenia. Neurosci Lett.

[11] Lawford BR, Young RM, Swagell CD, Barnes M, Burton SC, Ward WK, Heslop KR, Shadforth S, van Daal A, Morris CP (2005). The C/C genotype of the C957T polymorphism of the dopamine D2 receptor is associated with schizophrenia. Schizophr Res.

[12] Betcheva ET, Mushiroda T, Takahashi A, Kubo M, Karachanak SK, Zaharieva IT, Vazharova RV, Dimova II, Milanova VK, Tolev T (2009). Case–control association study of 59 candidate genes reveals the DRD2 SNP rs6277 (C957T) as the only susceptibility factor for schizophrenia in the Bulgarian population. J Hum Genet.

[13] Ponce G, Perez-Gonzalez R, Aragues M, Palomo T, Rodriguez-Jimenez R, Jimenez-Arriero MA, Hoenicka J (2009). The ANKK1 kinase gene and psychiatric disorders. Neurotox Res.

[14] Aslan S, Karaoguz MY, Eser HY, Karaer DK, Taner E (2010). Comparison of DRD2 rs1800497 (TaqIA) polymorphism between schizophrenic patients and healthy controls: lack of association in a Turkish sample. Int J Psychiatry Clin Pract.

[15] Itokawa M, Arinami T, Toru M (2010). Advanced research on dopamine signaling to develop drugs for the treatment of mental disorders: Ser311Cys polymorphisms of the dopamine D2-receptor gene and schizophrenia. J Pharmacol Sci.

[16] Kaneshima M, Higa T, Nakamoto H, Nagamine M (1997). An association study between the Cys311 variant of dopamine D2 receptor gene and schizophrenia in the Okinawan population. Psychiatry Clin Neurosci.

[17] Behravan J, Hemayatkar M, Toufani H, Abdollahian E (2008). Linkage and association of DRD2 gene TaqI polymorphism with schizophrenia in an Iranian population. Arch Iran Med.

[18] Jonsson EG, Sillen A, Vares M, Ekholm B, Terenius L, Sedvall GC (2003). Dopamine D2 receptor gene Ser311Cys variant and schizophrenia: association study and meta-analysis. Am J Med Genet B Neuropsychiatr Genet.

[19] Hoenicka J, Aragues M, Rodriguez-Jimenez R, Ponce G, Martinez I, Rubio G, Jimenez-Arriero MA, Palomo T (2006). C957T DRD2 polymorphism is associated with schizophrenia in Spanish patients. Acta Psychiatr Scand.

[20] Fan H, Zhang F, Xu Y, Huang X, Sun G, Song Y, Long H, Liu P (2010). An association study of DRD2 gene polymorphisms with schizophrenia in a Chinese Han population. Neurosci Lett.

[21] Yao J, Pan YQ, Ding M, Pang H, Wang BJ (2015). Association between DRD2 (rs1799732 and rs1801028) and ANKK1 (rs1800497) polymorphisms and schizophrenia: a meta-analysis. Am J Med Genet Part B Neuropsychiatr Genet.

[22] Liu L, Fan D, Ding N, Hu Y, Cai G, Wang L, Xin L, Xia Q, Li X, Xu S (2014). The relationship between DRD2 gene polymorphisms (C957T and C939T) and schizophrenia: a meta-analysis. Neurosci Lett.

[23] Swartz MK (2011). The PRISMA statement: a guideline for systematic reviews and meta-analyses. J Pediatr Health Care.

[24] Moher D, Liberati A, Tetzlaff J, Altman DG (2010). Preferred reporting items for systematic reviews and meta-analyses: the PRISMA statement. Int J Surg.

[25] Stang A (2010). Critical evaluation of the Newcastle–Ottawa scale for the assessment of the quality of nonrandomized studies in meta-analyses. Eur J Epidemiol.

[26] Itokawa M, Arinami T, Futamura N, Hamaguchi H, Toru M (1993). A structural polymorphism of human dopamine D2 receptor, D2(Ser311→Cys). Biochem Biophys Res Commun.

[27] Arinami T, Itokawa M, Enguchi H, Tagaya H, Yano S, Shimizu H, Hamaguchi H, Toru M (1994). Association of dopamine D2 receptor molecular variant with schizophrenia. Lancet.

[28] Hattori M, Nanko S, Dai XY, Fukuda R, Kazamatsuri H (1994). Mismatch PCR RFLP detection of DRD2 Ser311Cys polymorphism and schizophrenia. Biochem Biophys Res Commun.

[29] Nanko S, Hattori M, Dai XY, Fukuda R, Kazamatsuri H (1994). DRD2 Ser311/Cys311 polymorphism in schizophrenia. Lancet.

[30] Arinami T, Itokawa M, Aoki J, Shibuya H, Ookubo Y, Iwawaki A, Ota K, Shimizu H, Hamaguchi H, Toru M (1996). Further association study on dopamine D2 receptor variant S311C in schizophrenia and affective disorders. Am J Med Genet.

[31] Chen CH, Chien SH, Hwu HG (1996). No association of dopamine D2 receptor molecular variant Cys311 and schizophrenia in Chinese patients. Am J Med Genet.

[32] Ohara K, Nakamura Y, Xie DW, Ishigaki T, Deng ZL, Tani K, Zhang HY, Kondo N, Liu JC, Miyasato K (1996). Polymorphisms of dopamine D2-like (D2, D3, and D4) receptors in schizophrenia. Biol Psychiatry.

[33] Fujiwara Y, Yamaguchi K, Tanaka Y, Tomita H, Shiro Y, Kashihara K, Sato K, Kuroda S (1997). Polymorphism of dopamine receptors and transporter genes in neuropsychiatric diseases. Eur Neurol.

[34] Harano M (1997). Ser-311-Cys polymorphism of the dopamine D2 receptor gene and schizophrenia—an analysis of schizophrenic patients in Fukuoka. Kurume Med J.

[35] Tanaka T, Igarashi S, Onodera O, Tanaka H, Fukushima N, Takahashi M, Kameda K, Tsuji S, Ihda S (1996). Lack of association between dopamine D2 receptor gene Cys311 variant and schizophrenia. Am J Med Genet.

[36] Spurlock G, Williams J, McGuffin P, Aschauer HN, Lenzinger E, Fuchs K, Sieghart WC, Meszaros K, Fathi N, Laurent C (1998). European multicentre association study of schizophrenia: a study of the DRD2 Ser311Cys and DRD3 Ser9Gly polymorphisms. Am J Med Genet.

[37] Morimoto K, Miyatake R, Nakamura M, Watanabe T, Hirao T, Suwaki H (2002). Delusional disorder: molecular genetic evidence for dopamine psychosis. Neuropsychopharmacology.

[38] Serretti A, Lattuada E, Lorenzi C, Lilli R, Smeraldi E (2000). Dopamine receptor D2 Ser/Cys 311 variant is associated with delusion and disorganization symptomatology in major psychoses. Mol Psychiatry.

[39] Hori H, Ohmori O, Shinkai T, Kojima H, Nakamura J (2001). Association analysis between two functional dopamine D2 receptor gene polymorphisms and schizophrenia. Am J Med Genet.

[40] Himei A, Koh J, Sakai J, Inada Y, Akabame K, Yoneda H (2002). The influence on the schizophrenic symptoms by the DRD2Ser/Cys311 and −141C Ins/Del polymorphisms. Psychiatry Clin Neurosci.

[41] Gupta M, Chauhan C, Bhatnagar P, Gupta S, Grover S, Singh PK, Purushottam M, Mukherjee O, Jain S, Brahmachari SK (2009). Genetic susceptibility to schizophrenia: role of dopaminergic pathway gene polymorphisms. Pharmacogenomics.

[42] Tsutsumi A, Glatt SJ, Kanazawa T, Kawashige S, Uenishi H, Hokyo A, Kaneko T, Moritani M, Kikuyama H, Koh J (2011). The genetic validation of heterogeneity in schizophrenia. Behav Brain Funct.

[43] Lafuente A, Bernardo M, Mas S, Crescenti A, Aparici M, Gasso P, Deulofeu R, Mane A, Catalan R, Carne X (2008). Polymorphism of dopamine D2 receptor (TaqIA, TaqIB, and −141C Ins/Del) and dopamine degradation enzyme (COMT G158A, A-278G) genes and extrapyramidal symptoms in patients with schizophrenia and bipolar disorders. Psychiatry Res.

[44] Monakhov M, Golimbet V, Abramova L, Kaleda V, Karpov V (2008). Association study of three polymorphisms in the dopamine D2 receptor gene and schizophrenia in the Russian population. Schizophr Res.

[45] Lafuente A, Bernardo M, Mas S, Crescenti A, Aparici M, Gasso P, Goti J, Sanchez V, Catalan R, Carne X (2008). −141C Ins/Del polymorphism of the dopamine D2 receptor gene is associated with schizophrenia in a Spanish population. Psychiatr Genet.

[46] Dubertret C, Bardel C, Ramoz N, Martin PM, Deybach JC, Ades J, Gorwood P, Gouya L (2010). A genetic schizophrenia-susceptibility region located between the ANKK1 and DRD2 genes. Prog Neuropsychopharmacol Biol Psychiatry.

[47] Kukreti R, Tripathi S, Bhatnagar P, Gupta S, Chauhan C, Kubendran S, Janardhan Reddy YC, Jain S, Brahmachari SK (2006). Association of DRD2 gene variant with schizophrenia. Neurosci Lett.

[48] Mo GH, Lai IC, Wang YC, Chen JY, Lin CY, Chen TT, Chen ML, Liou YJ, Liao DL, Bai YM (2007). Support for an association of the C939T polymorphism in the human DRD2 gene with tardive dyskinesia in schizophrenia. Schizophr Res.

[49] Luo PF (2008). Association of dopamine D2 receptor polymorphisms with paranoid schizophrenia in the North Chinese population.

[50] Asherson P, Williams N, Roberts E, McGuffin M, Owen M (1994). DRD2 Ser311/Cys311 polymorphism in schizophrenia. Lancet.

[51] Crawford F, Hoyne J, Cai X, Osborne A, Poston D, Zaglul J, Dajani N, Walsh S, Bradley R, Solomon R (1996). Dopamine DRD2/Cys311 is not associated with chronic schizophrenia. Am J Med Genet.

[52] Dubertret C, Gouya L, Hanoun N, Deybach JC, Ades J, Hamon M, Gorwood P (2004). The 3′ region of the DRD2 gene is involved in genetic susceptibility to schizophrenia. Schizophr Res.

[53] Gejman PV, Ram A, Gelernter J, Friedman E, Cao Q, Pickar D, Blum K, Noble EP, Kranzler HR, O’Malley S (1994). No structural mutation in the dopamine D2 receptor gene in alcoholism or schizophrenia. Analysis using denaturing gradient gel electrophoresis. JAMA.

[54] Gonzalez-Castro TB, Tovilla-Zarate CA, Hernandez-Diaz Y, Fresan A, Juarez-Rojop IE, Ble-Castillo JL, Lopez-Narvaez L, Genis A, Hernandez-Alvarado MM (2015). No association between ApoE and schizophrenia: evidence of systematic review and updated meta-analysis. Schizophr Res.

[55] Laurent C, Bodeau-Pean S, Campion D, d’Amato T, Jay M, Dollfus S, Thibault F, Petit M, Samolyk D, Martinez M (1994). No major role for the dopamine D2 receptor Ser→Cys311 mutation in schizophrenia. Psychiatr Genet.

[56] Nothen MM, Wildenauer D, Cichon S, Albus M, Maier W, Minges J, Lichtermann D, Bondy B, Rietschel M, Korner J (1994). Dopamine D2 receptor molecular variant and schizophrenia. Lancet.

[57] Sanders AR, Duan J, Levinson DF, Shi J, He D, Hou C, Burrell GJ, Rice JP, Nertney DA, Olincy A (2008). No significant association of 14 candidate genes with schizophrenia in a large European ancestry sample: implications for psychiatric genetics. Am J Psychiatry.

[58] Sasaki T, Macciardi FM, Badri F, Verga M, Meltzer HY, Lieberman J, Howard A, Bean G, Joffe RT, Hudson CJ (1996). No evidence for association of dopamine D2 receptor variant (Ser311/Cys311) with major psychosis. Am J Med Genet.

[59] Shaikh S, Collier D, Arranz M, Ball D, Gill M, Kerwin R (1994). DRD2 Ser311/Cys311 polymorphism in schizophrenia. Lancet.

[60] Sobell J, Sigurdson DC, Heston L, Sommer S (1994). S311C D2DR variant: no association with schizophrenia. Lancet.

[61] Srivastava V, Deshpande SN, Thelma BK (2010). Dopaminergic pathway gene polymorphisms and genetic susceptibility to schizophrenia among north Indians. Neuropsychobiology.

[62] Verga M, Macciardi F, Pedrini S, Cohen S, Smeraldi E (1997). No association of the Ser/Cys311 DRD2 molecular variant with schizophrenia using a classical case control study and the haplotype relative risk. Schizophr Res.

[63] Comings DE, Comings BG, Muhleman D, Dietz G, Shahbahrami B, Tast D, Knell E, Kocsis P, Baumgarten R, Kovacs BW (1991). The dopamine D2 receptor locus as a modifying gene in neuropsychiatric disorders. JAMA.

[64] Stober G, Jatzke S, Heils A, Jungkunz G, Knapp M, Mossner R, Riederer P, Lesch KP (1998). Insertion/deletion variant (−141C Ins/Del) in the 5′ regulatory region of the dopamine D2 receptor gene: lack of association with schizophrenia and bipolar affective disorder. Short communication. J Neural Transm.

[65] Sanders AR, Rincon-Limas DE, Chakraborty R, Grandchamp B, Hamilton JD, Fann WE, Patel PI (1993). Association between genetic variation at the porphobilinogen deaminase gene and schizophrenia. Schizophr Res.

[66] Jonsson EG, Nothen MM, Neidt H, Forslund K, Rylander G, Mattila-Evenden M, Asberg M, Propping P, Sedvall GC (1999). Association between a promoter polymorphism in the dopamine D2 receptor gene and schizophrenia. Schizophr Res.

[67] Luu SU, Liao HM, Hung TW, Liu BY, Cheng MC, Liao DL, Chen SJ, Chen CH (2008). Mutation analysis of adenosine A2a receptor gene and interaction study with dopamine D2 receptor gene in schizophrenia. Psychiatr Genet.

[68] Cordeiro Q, Siqueira-Roberto J, Zung S, Vallada H (2009). Association between the DRD2 −141C insertion/deletion polymorphism and schizophrenia. Arq Neuropsiquiatr.

[69] Xiao L, Shen T, Peng DH, Shu C, Jiang KD, Wang GH (2013). Functional −141C Ins/Del polymorphism in the dopamine D2 receptor gene promoter and schizophrenia in a Chinese Han population. J Int Med Res.

[70] Gejman PV, Sanders AR, Duan J (2010). The role of genetics in the etiology of schizophrenia. Psychiatr Clin N Am.

[71] Schwab SG, Wildenauer DB (2013). Genetics of psychiatric disorders in the GWAS era: an update on schizophrenia. Eur Arch Psychiatry Clin Neurosci.

[72] Gonzalez-Castro TB, Tovilla-Zarate CA (2014). Meta-analysis: a tool for clinical and experimental research in psychiatry. Nord J Psychiatry.

[73] Li W, Guo X, Xiao S (2015). Evaluating the relationship between reelin gene variants (rs7341475 and rs262355) and schizophrenia: a meta-analysis. Neurosci Lett.

[74] Comings DE, Muhleman D, Ahn C, Gysin R, Flanagan SD (1994). The dopamine D2 receptor gene: a genetic risk factor in substance abuse. Drug Alcohol Depend.

[75] Blum K, Braverman ER, Wood RC, Gill J, Li C, Chen TJ, Taub M, Montgomery AR, Sheridan PJ, Cull JG (1996). Increased prevalence of the Taq I A1 allele of the dopamine receptor gene (DRD2) in obesity with comorbid substance use disorder: a preliminary report. Pharmacogenetics.

[76] Suzuki A, Mihara K, Kondo T, Tanaka O, Nagashima U, Otani K, Kaneko S (2000). The relationship between dopamine D2 receptor polymorphism at the Taq1 A locus and therapeutic response to nemonapride, a selective dopamine antagonist, in schizophrenic patients. Pharmacogenetics.

[77] Mihara K, Suzuki A, Kondo T, Nagashima U, Ono S, Otani K, Kaneko S (2000). No relationship between Taq1 a polymorphism of dopamine D(2) receptor gene and extrapyramidal adverse effects of selective dopamine D(2) antagonists, bromperidol, and nemonapride in schizophrenia: a preliminary study. Am J Med Genet.

[78] Noble EP (1998). The D2 dopamine receptor gene: a review of association studies in alcoholism and phenotypes. Alcohol.

[79] Campion D, d’Amato T, Bastard C, Laurent C, Guedj F, Jay M, Dollfus S, Thibaut F, Petit M, Gorwood P (1994). Genetic study of dopamine D1, D2, and D4 receptors in schizophrenia. Psychiatry Res.

[80] Dollfus S, Campion D, Vasse T, Preterre P, Laurent C, d’Amato T, Thibaut F, Mallet J, Petit M (1996). Association study between dopamine D1, D2, D3, and D4 receptor genes and schizophrenia defined by several diagnostic systems. Biol Psychiatry.

[81] Parsons MJ, Mata I, Beperet M, Iribarren-Iriso F, Arroyo B, Sainz R, Arranz MJ, Kerwin R (2007). A dopamine D2 receptor gene-related polymorphism is associated with schizophrenia in a Spanish population isolate. Psychiatr Genet.

[82] Li T, Arranz M, Aitchison KJ, Bryant C, Liu X, Kerwin RW, Murray R, Sham P, Collier DA (1998). Case–control, haplotype relative risk and transmission disequilibrium analysis of a dopamine D2 receptor functional promoter polymorphism in schizophrenia. Schizophr Res.

[83] Breen G, Brown J, Maude S, Fox H, Collier D, Li T, Arranz M, Shaw D, StClair D (1999). −141 C del/ins polymorphism of the dopamine receptor 2 gene is associated with schizophrenia in a British population. Am J Med Genet.

[84] Inada T, Arinami T, Yagi G (1999). Association between a polymorphism in the promoter region of the dopamine D2 receptor gene and schizophrenia in Japanese subjects: replication and evaluation for antipsychotic-related features. Int J Neuropsychopharmacol.

[85] Tallerico T, Ulpian C, Liu IS (1999). Dopamine D2 receptor promoter polymorphism: no association with schizophrenia. Psychiatry Res.

[86] Kampman O, Anttila S, Illi A, Lehtimaki T, Mattila KM, Roivas M, Leinonen E (2003). Dopamine receptor D2 −141C insertion/deletion polymorphism in a Finnish population with schizophrenia. Psychiatry Res.

[87] Kurt H, Dikmen M, Basaran A, Yenilmez C, Ozdemir F, Degirmenci I, Gunes HV, Kucuk MU, Mutlu F (2011). Dopamine D2 receptor gene −141C insertion/deletion polymorphism in Turkish schizophrenic patients. Mol Biol Rep.

[88] Saiz PA, Garcia-Portilla MP, Arango C, Morales B, Arias B, Corcoran P, Fernandez JM, Alvarez V, Coto E, Bascaran MT (2010). Genetic polymorphisms in the dopamine-2 receptor (DRD2), dopamine-3 receptor (DRD3), and dopamine transporter (SLC6A3) genes in schizophrenia: data from an association study. Prog Neuropsychopharmacol Biol Psychiatry.

