# Supplementary material for: The role of C957T, TaqI and Ser311Cys polymorphisms of the DRD2 gene in schizophrenia: systematic review and meta-analysis
Source: Behav Brain Funct. 2016 Nov 9;12:29. doi: 10.1186/s12993-016-0114-z (PMC5101652; doi:10.1186/s12993-016-0114-z)
Supplement: Supplementary file 1 — Additional file 1. Grades quality assessment and PRISMA check list. [file 12993_2016_114_MOESM1_ESM.pdf]

Section/topic	#	Checklist item	Reported on page #
**TITLE**			
Title	1	Identify the report as a systematic review, meta-analysis, or both.	1
**ABSTRACT**			
Structured summary	2	Provide a structured summary including, as applicable: background; objectives; data sources; study eligibility criteria, participants, and interventions; study appraisal and synthesis methods; results; limitations; conclusions and implications of key findings; systematic review registration number.	2
**INTRODUCTION**			
Rationale	3	Describe the rationale for the review in the context of what is already known.	3
Objectives	4	Provide an explicit statement of questions being addressed with reference to participants, interventions, comparisons, outcomes, and study design (PICOS).	4
**METHODS**			
Protocol and registration	5	Indicate if a review protocol exists, if and where it can be accessed (e.g., Web address), and, if available, provide registration information including registration number.	5
Eligibility criteria	6	Specify study characteristics (e.g., PICOS, length of follow-up) and report characteristics (e.g., years considered, language, publication status) used as criteria for eligibility, giving rationale.	5
Information sources	7	Describe all information sources (e.g., databases with dates of coverage, contact with study authors to identify additional studies) in the search and date last searched.	5
Search	8	Present full electronic search strategy for at least one database, including any limits used, such that it could be repeated.	5
Study selection	9	State the process for selecting studies (i.e., screening, eligibility, included in systematic review, and, if applicable, included in the meta-analysis).	6
Data collection process	10	Describe method of data extraction from reports (e.g., piloted forms, independently, in duplicate) and any processes for obtaining and confirming data from investigators.	6
Data items	11	List and define all variables for which data were sought (e.g., PICOS, funding sources) and any assumptions and simplifications made.	6
Risk of bias in individual studies	12	Describe methods used for assessing risk of bias of individual studies (including specification of whether this was done at the study or outcome level), and how this information is to be used in any data synthesis.	6-7
Summary measures	13	State the principal summary measures (e.g., risk ratio, difference in means).	7
Synthesis of results	14	Describe the methods of handling data and combining results of studies, if done, including measures of consistency (e.g., I^2) for each meta-analysis.	8

Section/topic	#	Checklist item	Reported on page #
Risk of bias across studies	15	Specify any assessment of risk of bias that may affect the cumulative evidence (e.g., publication bias, selective reporting within studies).	8
Additional analyses	16	Describe methods of additional analyses (e.g., sensitivity or subgroup analyses, meta-regression), if done, indicating which were pre-specified.	7-8
**RESULTS**			
Study selection	17	Give numbers of studies screened, assessed for eligibility, and included in the review, with reasons for exclusions at each stage, ideally with a flow diagram.	8-9
Study characteristics	18	For each study, present characteristics for which data were extracted (e.g., study size, PICOS, follow-up period) and provide the citations.	Table 3
Risk of bias within studies	19	Present data on risk of bias of each study and, if available, any outcome level assessment (see item 12).	9-13
Results of individual studies	20	For all outcomes considered (benefits or harms), present, for each study: (a) simple summary data for each intervention group (b) effect estimates and confidence intervals, ideally with a forest plot.	9-13
Synthesis of results	21	Present results of each meta-analysis done, including confidence intervals and measures of consistency.	Table 4-6
Risk of bias across studies	22	Present results of any assessment of risk of bias across studies (see Item 15).	Table 4-6
Additional analysis	23	Give results of additional analyses, if done (e.g., sensitivity or subgroup analyses, meta-regression [see Item 16]).	13<br>Table 1-2
**DISCUSSION**			
Summary of evidence	24	Summarize the main findings including the strength of evidence for each main outcome; consider their relevance to key groups (e.g., healthcare providers, users, and policy makers).	14-15
Limitations	25	Discuss limitations at study and outcome level (e.g., risk of bias), and at review-level (e.g., incomplete retrieval of identified research, reporting bias).	16
Conclusions	26	Provide a general interpretation of the results in the context of other evidence, and implications for future research.	16
**FUNDING**			
Funding	27	Describe sources of funding for the systematic review and other support (e.g., supply of data); role of funders for the systematic review.	17

From: Moher D, Liberati A, Tetzlaff J, Altman DG, The PRISMA Group (2009). Preferred Reporting Items for Systematic Reviews and Meta-Analyses: The PRISMA Statement. PLoS Med 6(6): e1000097. doi:10.1371/journal.pmed1000097

Table 1. Summary of the findings of association studies on the DRD2 gene and SZ risk.

Reference	OR (95% CI)	Number of patients (studies)	Design	Quality evidence (GRADE)	Publication bias
Itokawa, M. [24]	2.26 (0.06-8.00)	50	Case-control	⊕⊕⊕⊕ High	Undetected
Arinami, T. [25]	3.06 (1.42-6.67)	156	Case-control	⊕⊕⊕⊕ High	Undetected
Hattori, M. [26]	2.04 (0.60-6.89)	100	Case-control	⊕⊕⊕○ Moderate	Undetected
Nanko, S. [27]	2.04 (0.60-6.89)	100	Case-control	⊕⊕⊕○ Moderate	Undetected
Arinami, T. [28]	1.06 (0.56-1.13)	136	Case-control	⊕⊕⊕○ Moderate	Undetected
Chen, C. H. [29]	1.78 (0.42-7.56)	114	Case-control	⊕⊕⊕○ Moderate	Undetected
Ohara, K. [30]	1.03 (0.88-1.20)	153	Case-control	⊕⊕⊕○ Moderate	Undetected
Fujiwara, Y. [31]	0.47 (0.09-2.28)	106	Case-control	⊕⊕⊕○ Moderate	Undetected
Tanaka, T. [33]	1.00 (0.24-4.10)	52	Case-control	⊕⊕⊕⊕ High	Undetected
Kaneshima, M. [16]	0.81 (0.23-2.83)	78	Case-control	⊕⊕⊕⊕ High	Undetected
Harano, M. [32]	1.47 (0.53-4.01)	70	Case-control	⊕⊕⊕○ Moderate	Undetected
Spurlock, G. [34]	0.91 (0.44-1.85)	373	Case-control	⊕⊕⊕○ Moderate	Undetected
Serreti, A. [36]	0.54 (0.29-1.00)	366	Case-control	⊕⊕⊕⊕ High	Undetected
Hori, H. [37]	1.23 (0.65-2.32)	241	Case-control	⊕⊕⊕○ Moderate	Undetected
Himei, A. [38]	1.37 (0.52-3.58)	190	Case-control	⊕⊕⊕○ Moderate	Undetected
Morimoto, K. [35]	1.00 (0.19-5.08)	48	Case-control	⊕⊕⊕○ Moderate	Undetected
Asherson, P.[48]	1.13(0.10-12.64)	112	Case-control	⊕⊕⊕○ Moderate	Undetected
Gejman, P.V. [51]	0.79(0.17-3.60)	102	Case-control	⊕⊕⊕⊕ High	Undetected
Laurent, C. [53]	2.75(0.65-11.63)	113	Case-control	⊕⊕⊕○ Moderate	Undetected
Nöthen, M. [54]	0.61(0.16-2.30)	179	Case-control	⊕⊕⊕⊕ High	Undetected
Shaikh, S. [57]	4.85(0.59-39.75)	147	Case-control	⊕⊕⊕○ Moderate	Undetected
Sobell, J. [58]	1.18(0.66-2.12)	338	Case-control	⊕⊕⊕⊕ High	Undetected
Sasaki, T. [56]	1.01(0.44-2.33)	273	Case-control	⊕⊕⊕○ Moderate	Undetected
Crawford, F. [49]	2.30(0.58-9.07)	84	Case-control	⊕⊕⊕○ Moderate	Undetected
Verga, M. [60]	2.13(0.72-6.25)	103	Case-control	⊕⊕⊕○ Moderate	Undetected
Dubertret, C. [50]	1.63(0.40-6.61)	103	Case-control	⊕⊕⊕○ Moderate	Undetected
Sanders, A. [55]	0.73(0.54-0.98)	2002	Case-control	⊕⊕⊕○ Moderate	Undetected

Srivastava, V. [59]	0.90(0.55-1.46)	233	Case-control	⊕⊕⊕○ Moderate	Undetected
Itokawa, M. [15]	2.11(1.19-3.76)	280	Case-control	⊕⊕⊕○ Moderate	Undetected
Dubertret, C. [44]	0.98(0.28-3.44)	144	Case-control	⊕⊕⊕○ Moderate	Undetected
Jonsson, E. G. [18]	1.08 (0.88-1.33)	173	Case-control	⊕⊕⊕○ Moderate	Undetected
Vijayan, N. N. [1]	1.02 (0.65-1.60)	210	Case-control	⊕⊕⊕⊕ High	Undetected
Gupta, M. [39]	1.08 (0.70-1.68)	48	Case-control	⊕⊕⊕○ Moderate	Undetected
Fan, H. [20]	1.26 (0.75-2.12)	420	Case-control	⊕⊕⊕⊕ High	Undetected
Tsutsumi, A. [40]	0.74 (0.45-1.22)	406	Case-control	⊕⊕⊕○ Moderate	Undetected
Lafuente, A. [41]	0.98 (0.61-1.56)	80	Case-control	⊕⊕⊕⊕ High	Undetected
Monakhov, M. [42]	0.79 (0.60-1.03)	311	Case-control	⊕⊕⊕○ Moderate	Undetected
Lafuente, A. [43]	0.91 (0.67-1.23)	287	Case-control	⊕⊕⊕○ Moderate	Undetected
Behravan, J. [17]	1.38 (0.77-2.47)	38	Case-control	⊕⊕⊕○ Moderate	Undetected
Dubertret, C. [44]	1.44 (0.96-2.15)	144	Case-control	⊕⊕⊕○ Moderate	Undetected
Aslan, S. [14]	1.10 (0.74-1.61)	99	Case-control	⊕⊕⊕○ Moderate	Undetected
Lawford, B.R. [11]	1.73 (1.25-2.39)	154	Case-control	⊕⊕⊕○ Moderate	Undetected
Hanninen, K. [10]	1.26 (0.98-1.62)	188	Case-control	⊕⊕⊕○ Moderate	Undetected
Kukreti, R. [45]	1.18 (0.82-1.71)	101	Case-control	⊕⊕⊕○ Moderate	Undetected
Hoenicka, J. [19]	1.49 (1.12-1.98)	131	Case-control	⊕⊕⊕⊕ High	Undetected
Mo, G.H. [46]	1.67 (1.20-2.32)	174	Case-control	⊕⊕⊕○ Moderate	Undetected
Luo, F. P.[47]	1.79 (0.65-2.32)	466	Case-control	⊕⊕⊕○ Moderate	Undetected
Betcheva, E. T. [12]	1.26 (0.75-2.12)	255	Case-control	⊕⊕⊕○ Moderate	Undetected
