# Supplementary material for: The role of C957T, TaqI and Ser311Cys polymorphisms of the DRD2 gene in schizophrenia: systematic review and meta-analysis
Source: Behav Brain Funct. 2016 Nov 9;12:29. doi: 10.1186/s12993-016-0114-z (PMC5101652; doi:10.1186/s12993-016-0114-z)

Table S5. Characteristics of the association studies of Ser311Cys and SZ.

Author	Location	NOS	Number		Males %		Mean age		Genotypes						P for HWE	
			Cases	Controls	Cases	Controls	Cases	Controls	Cases			Controls			Cases	Controls
									Ser/Ser	Ser/Cys	Cys/Cys	Ser/Ser	Ser/Cys	Cys/Cys		
**Ser311Cys**																
Itokawa, M. [26]	Japan	5	50	110	-	-	-	-	46	3	1	105	5	0	0.09	0.93
Asherson, P.[50]	England	5	112	64	-	-	-	-	110	2	0	63	1	0	0.89	0.92
Gejman, P.V. [53]	USA	5	102	113		-	-	-	103	3	0	109	4	0	0.83	0.78
Laurent, C. [55]	France	5	113	184	-	-	-	-	108	5	0	181	3	0	0.73	0.87
Nöthen, M. [56]	Germany	5	179	138	-	-	-	-	175	4	0	133	5	0	0.83	0.76
Shaikh, S. [59]	England	6	147	100	-	-	-	-	140	7	0	99	1	0	0.67	0.94
Sobell, J. [60]	USA	6	338	1914	-	-	-	-	325	12	1	1848	65	1	0.12	0.44
Arinami, T. [27]	Japan	5	156	300	-	-	-	-	142	11	3	289	11	0	0.36	1.00
Hattori, M. [28]	Japan	5	100	100	-	-	-	-	92	8	0	96	4	0	0.98	1.00
Nanko, S. [29]	Japan	6	100	100	-	-	-	-	92	8	0	96	4	0	0.99	0.90
Sasaki, T. [58]	USA and Europe	6	273	255	-	-	32	36	261	12	0	245	9	1	0.60	0.10
Crawford, F. [51]	USA	6	84	81	-	-	-	-	78	5	1	78	3	0	0.12	0.81
Arinami, T. [30]	Japan	6	136	274	-	-	-	-	124	12	0	266	13	0	0.98	0.95
Chen, C. H. [31]	China	6	114	88	-	-	-	-	109	5	0	118	3	0	0.90	0.99
Ohara, K. [32]	Japan	6	153	121	50	-	26.3	-	152	1	0	98	8	0	0.97	1.00
Tanaka, T. [35]	Japan	6	106	106	51	59	-	-	97	9	0	98	8	0	1.00	1.00
Verga, M. [62]	Italy	7	103	97	65	-	37.9	53.25	92	11	0	92	5	0	0.43	0.71
Fujiwara, Y. [33]	Japan	6	52	26	-	-	-	-	50	2	0	93	8	0	1.00	0.94
Kaneshima, M. [16]	Japan	7	78	112	44	32	36.3	23	74	4	0	105	7	0	0.92	0.99
Harano, M. [34]	Japan	6	70	101	65	45	47	67	62	8	0	93	8	0	1.00	1.00
Spurlock, G. [36]	Austria, Ireland, Sweden, Italy and Wales	7	373	413	-	-	-	-	359	14	0	396	17	0	1.00	1.00
Serreti, A. [38]	Italy	7	366	267	44	48	44.7	46.6	329	37	0	252	15	0	0.61	1.00
Hori, H. [39]	Japan	7	241	201	51	49	52.7	54.7	217	23	1	185	15	1	0.48	0.29
Himei, A. [40]	Japan	7	190	103	63	51	53.1	33.3	175	15	0	97	6	0	1.00	0.96
Morimoto, K. [37]	Japan	7	48	48	-	-	0	0	45	3	0	45	3	0	0.99	1.00
Jonsson, E. G. [18]	Sweden	7	173	236	63	63	44.8	40	160	12	1	232	4	0	0.23	1.00
Dubertret, C. [52]	France	7	103	83	-	-	-	-	97	6	0	80	3	0	0.67	0.81
Vijayan, N. N. [1]	India	8	210	195	39	-	34.4	-	169	38	3	159	32	4	0.47	0.12
Sanders, A. [57]	USA	8	1870	2002	-	-	-	-	1796	73	1	1895	105	2	0.53	0.65
Gupta, M. [41]	India	8	48	48	-	-	-	-	208	42	4	186	37	2	0.27	0.70
Srivastava, V. [61]	India	8	233	224	-	-	21.4	34.6	200	32	1	189	34	1	0.81	0.67
Fan, H. [20]	China	8	420	403	-	-	30.7	28.1	387	32	1	377	26	0	0.50	1.00
Itokawa, M.[15]	Japan	8	156	300	-	-	-	-	142	11	3	289	11	0	0.00*	0.65
Itokawa, M. [15]	Japan	8	280	579	-	-	-	-	256	24	0	555	24	0	0.29	0.47
Dubertret, C. [46]	France	8	144	142	-	-	-	-	139	5	0	137	5	0	0.76	0.76
Tsutsumi, A. [42]	Japan	9	406	386	54	50	47.2	42.1	390	14	8	354	23	7	0.00*	0.00*

Table S6. Analysis of association studies on the Ser311Cys polymorphism of the DRD2 gene and schizophrenia by populations.

Model analysis		Model effects		P value of Q test	P value of Egger's test
		**Random** OR (95% CI)	**Fixed** OR (95% CI)		
**All populations**					
Allelic	With heterogeneity	**1.23(1.02-1.48)**	1.11(0.98-1.26)	0.004	0.049
	Without heterogeneity	0.89(0.75-1.06)	0.89(0.76-1.04)	0.255	0.758
Additive	With heterogeneity				
	Without heterogeneity	1.41(0.80-2.50)	1.41(0.80-2.50)	0.671	0.145
Recessive	With heterogeneity				
	Without heterogeneity	**2.36(1.40-3.98)**	**2.36(1.40-3.98)**	0.529	0.685
Dominant	With heterogeneity	**1.26(1.06-1.50)**	**1.16(1.02-1.32)**	0.031	0.045
	Without heterogeneity	**1.20(1.03-1.39)**	**1.20(1.03-1.39)**	0.807	0.085
**Caucasian population**					
Allelic	With heterogeneity				
	Without heterogeneity	1.11(0.82-1.49)	0.93(0.77-1.13)	0.061	0.098
Additive	With heterogeneity				
	Without heterogeneity	1.22(0.36-4.06)	1.22(0.36-4.06)	0.558	0.835
Recessive	With heterogeneity				
	Without heterogeneity	1.20(0.36-4.01)	1.20(0.36-4.01)	0.568	0.854
Dominant	With heterogeneity				
	Without heterogeneity	1.27(0.94-1.70)	1.06(0.87-1.29)	0.089	0.099
**Asian population**					
Allelic	With heterogeneity	**1.35(1.07-1.72)**	**1.30(1.10-1.54)**	0.040	0.559
	Without heterogeneity	**1.21(1.00-1.45)**	**1.20(1.00-1.45)**	0.439	0.218
Additive	With heterogeneity				
	Without heterogeneity	1.52(0.78-2.98)	1.51(0.78-2.94)	0.421	0.100
Recessive	With heterogeneity				
	Without heterogeneity	1.51(0.78-2.94)	1.51(0.78-2.94)	0.439	0.096
Dominant	With heterogeneity				
	Without heterogeneity	**1.32(1.05-1.65)**	**1.29(1.08-1.54)**	0.107	0.936

Figure S6. A) Forest plots of allelic model Ser311Cys. **B)** Begg's funnel plot analysis of publication bias for allelic model Ser311Cys. **C)** Forest plots of dominant model Ser311Cys. **D)** Begg's funnel plot analysis of publication bias for dominant model Ser311Cys.(without heterogeneity)

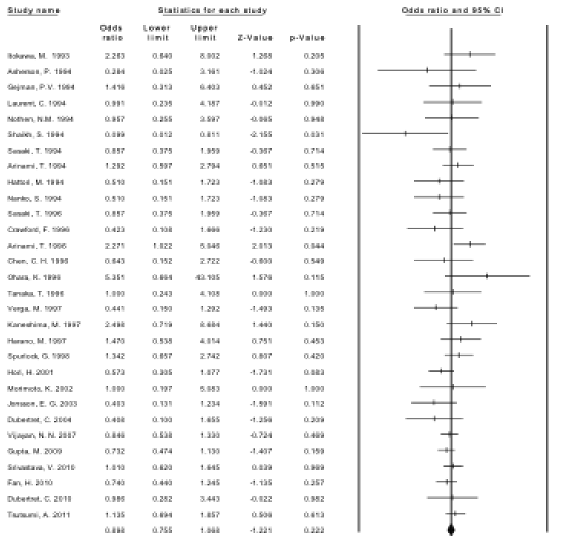

A)

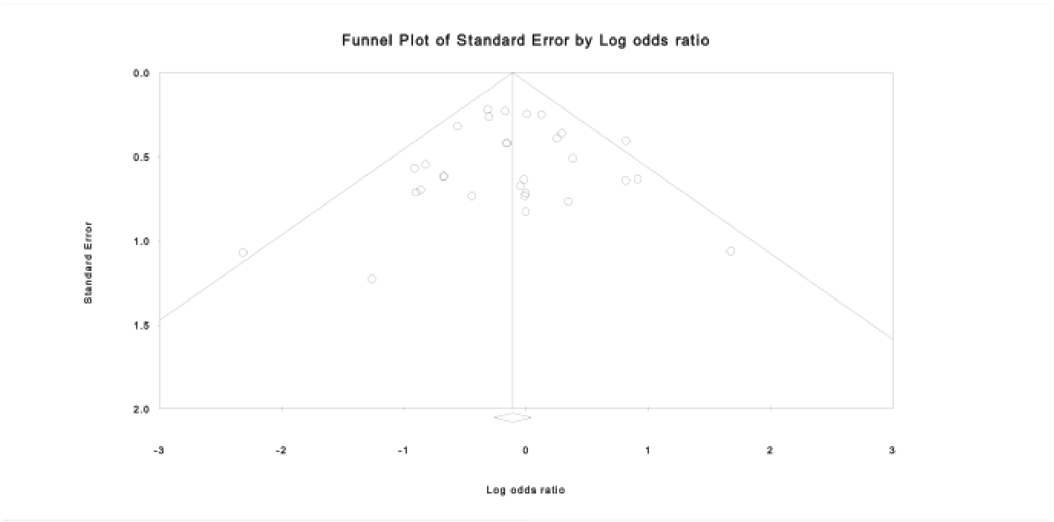

B)

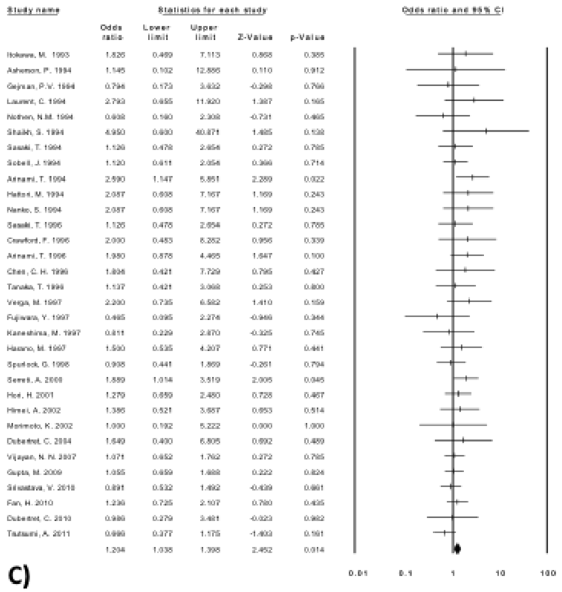

C)

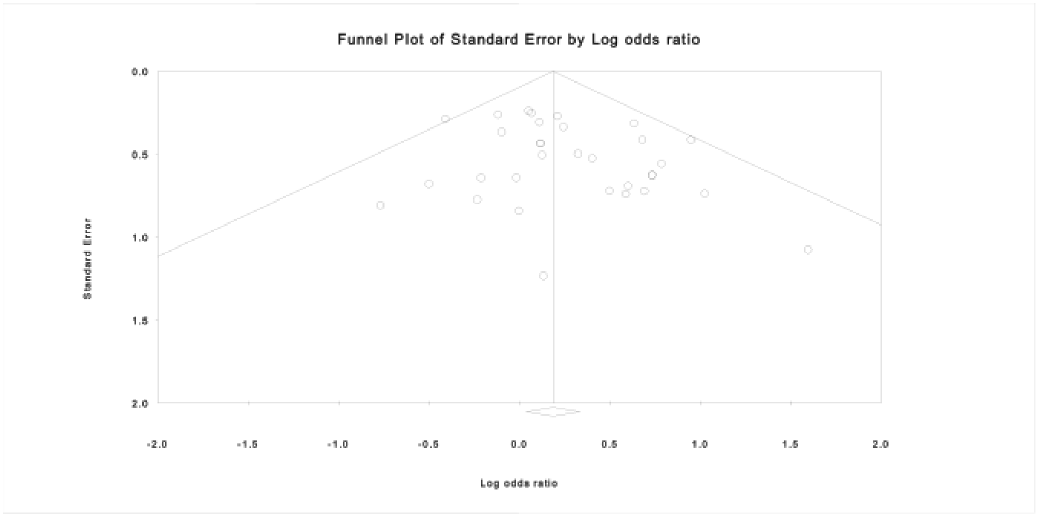

D)

Figure S7. A) Forest plots of allelic model Ser311Cys in Caucasians. **B)** Begg's funnel plot analysis of publication bias for allelic model Ser311Cys in Caucasians. **C)** Forest plots of dominant model Ser311Cys in Caucasians. **D)** Begg's funnel plot analysis of publication bias for dominant model Ser311Cys in Caucasians. (without heterogeneity)

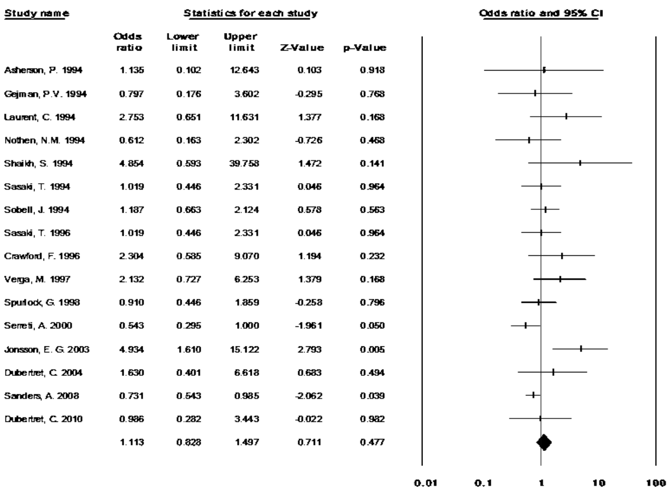

Study name	Odds ratio	Lower limit	Upper limit	Z-Value	p-Value
Asherson, P. 1994	1.135	0.102	12.643	0.103	0.918
Gejman, P.V. 1994	0.797	0.176	3.602	-0.295	0.768
Laurent, C. 1994	2.753	0.651	11.631	1.377	0.168
Nothen, N.M. 1994	0.612	0.163	2.302	-0.726	0.468
Shaikh, S. 1994	4.854	0.593	39.758	1.472	0.141
Sasaki, T. 1994	1.019	0.446	2.331	0.046	0.964
Sobell, J. 1994	1.187	0.663	2.124	0.578	0.563
Sasaki, T. 1996	1.019	0.446	2.331	0.046	0.964
Crawford, F. 1996	2.304	0.585	9.070	1.194	0.232
Verga, M. 1997	2.132	0.727	6.253	1.379	0.168
Spurlock, G. 1998	0.910	0.446	1.859	-0.258	0.796
Serreti, A. 2000	0.543	0.295	1.000	-1.961	0.050
Jonsson, E. G. 2003	4.934	1.610	15.122	2.793	0.005
Dubertret, C. 2004	1.630	0.401	6.618	0.683	0.494
Sanders, A. 2008	0.731	0.543	0.985	-2.062	0.039
Dubertret, C. 2010	0.986	0.282	3.443	-0.022	0.982
	1.113	0.828	1.497	0.711	0.477

A)

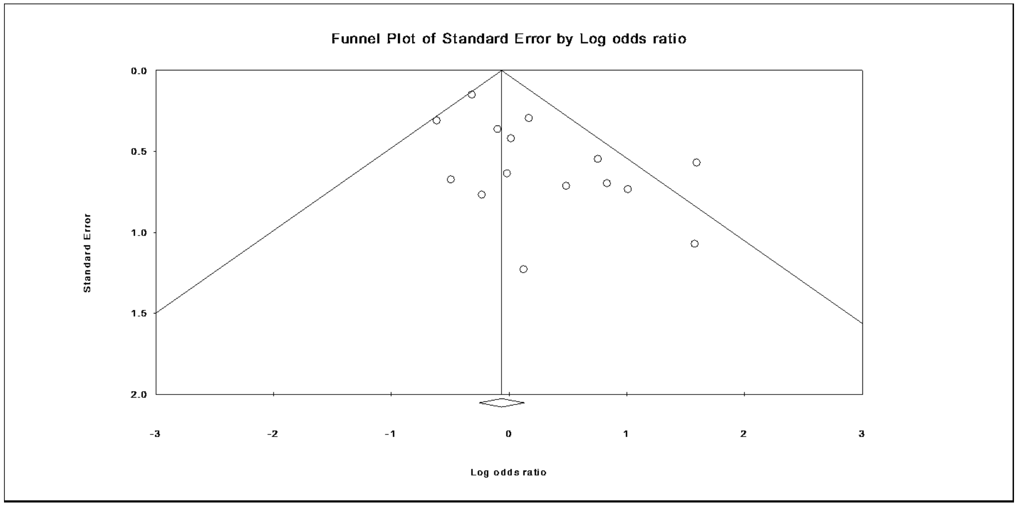

B)

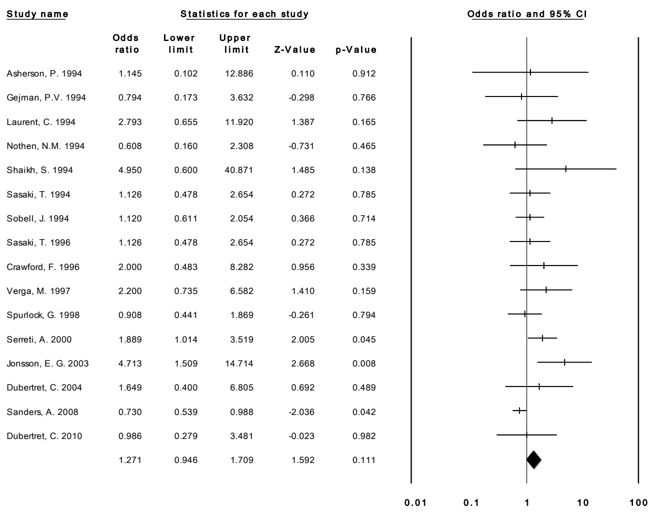

Study name	Odds ratio	Lower limit	Upper limit	Z-Value	p-Value
Asherson, P. 1994	1.145	0.102	12.886	0.110	0.912
Gejman, P.V. 1994	0.794	0.173	3.632	-0.298	0.766
Laurent, C. 1994	2.793	0.655	11.920	1.387	0.165
Nothen, N.M. 1994	0.608	0.160	2.308	-0.731	0.465
Shaikh, S. 1994	4.950	0.600	40.871	1.485	0.138
Sasaki, T. 1994	1.126	0.478	2.654	0.272	0.785
Sobell, J. 1994	1.120	0.611	2.054	0.366	0.714
Sasaki, T. 1996	1.126	0.478	2.654	0.272	0.785
Crawford, F. 1996	2.000	0.483	8.282	0.956	0.339
Verga, M. 1997	2.200	0.735	6.582	1.410	0.159
Spurlock, G. 1998	0.908	0.441	1.869	-0.261	0.794
Serreti, A. 2000	1.889	1.014	3.519	2.005	0.045
Jonsson, E. G. 2003	4.713	1.509	14.714	2.668	0.008
Dubertret, C. 2004	1.649	0.400	6.805	0.692	0.489
Sanders, A. 2008	0.730	0.539	0.988	-2.036	0.042
Dubertret, C. 2010	0.986	0.279	3.481	-0.023	0.982
	1.271	0.946	1.709	1.592	0.111

C)

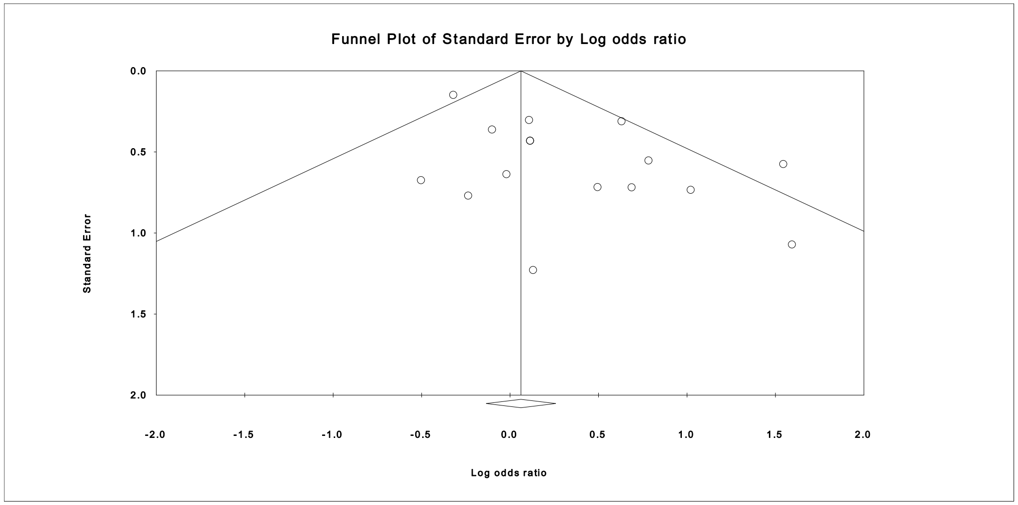

D)

Figure S8. A) Forest plots of allelic model Ser311Cys in Asians. **B)** Begg's funnel plot analysis of publication bias for allelic model Ser311Cys in Asians. **C)** Forest plots of dominant model Ser311Cys in Asians. **D)** Begg's funnel plot analysis of publication bias for dominant model Ser311Cys in Asians. .(without heterogeneity)

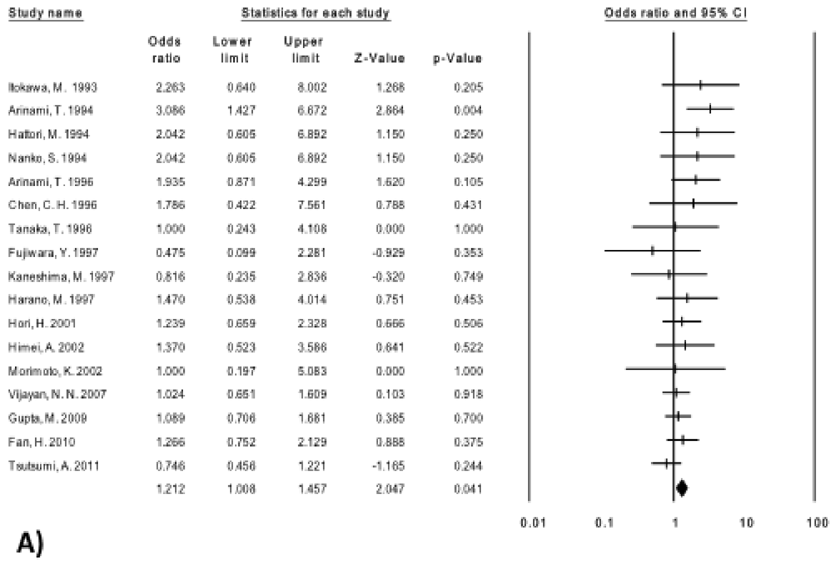

A)

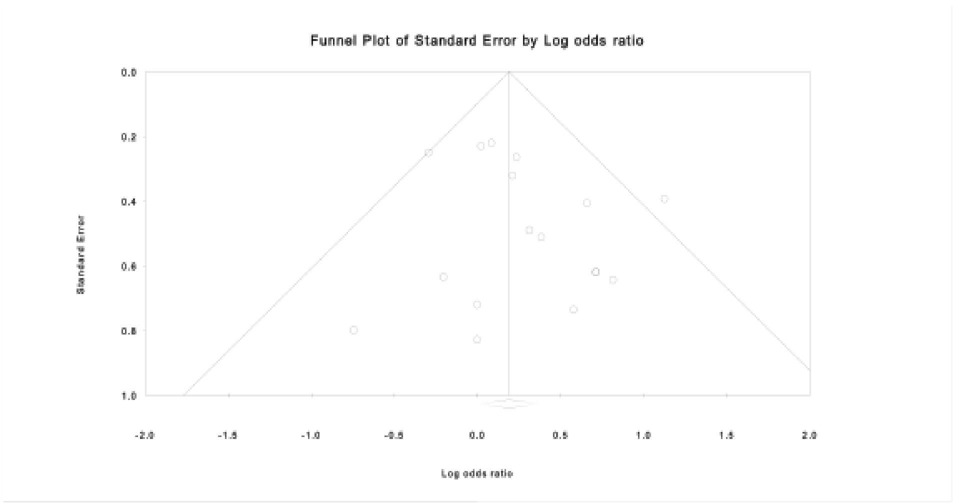

B)

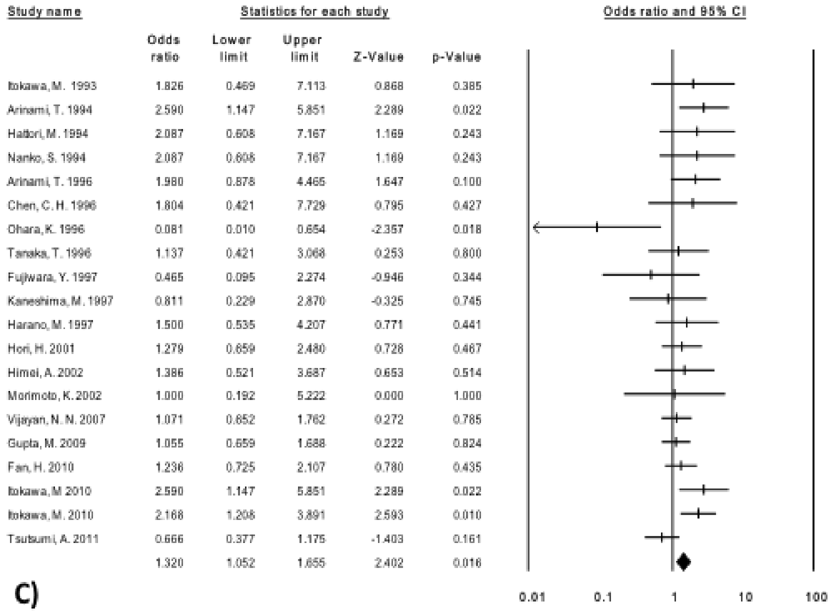

C)

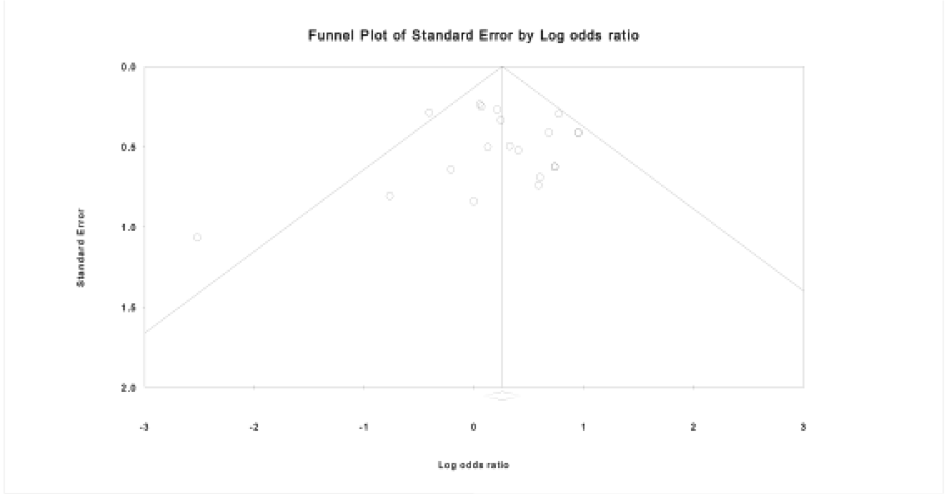

D)

Figure S9. Meta-regression based on ages of Ser311Cys.

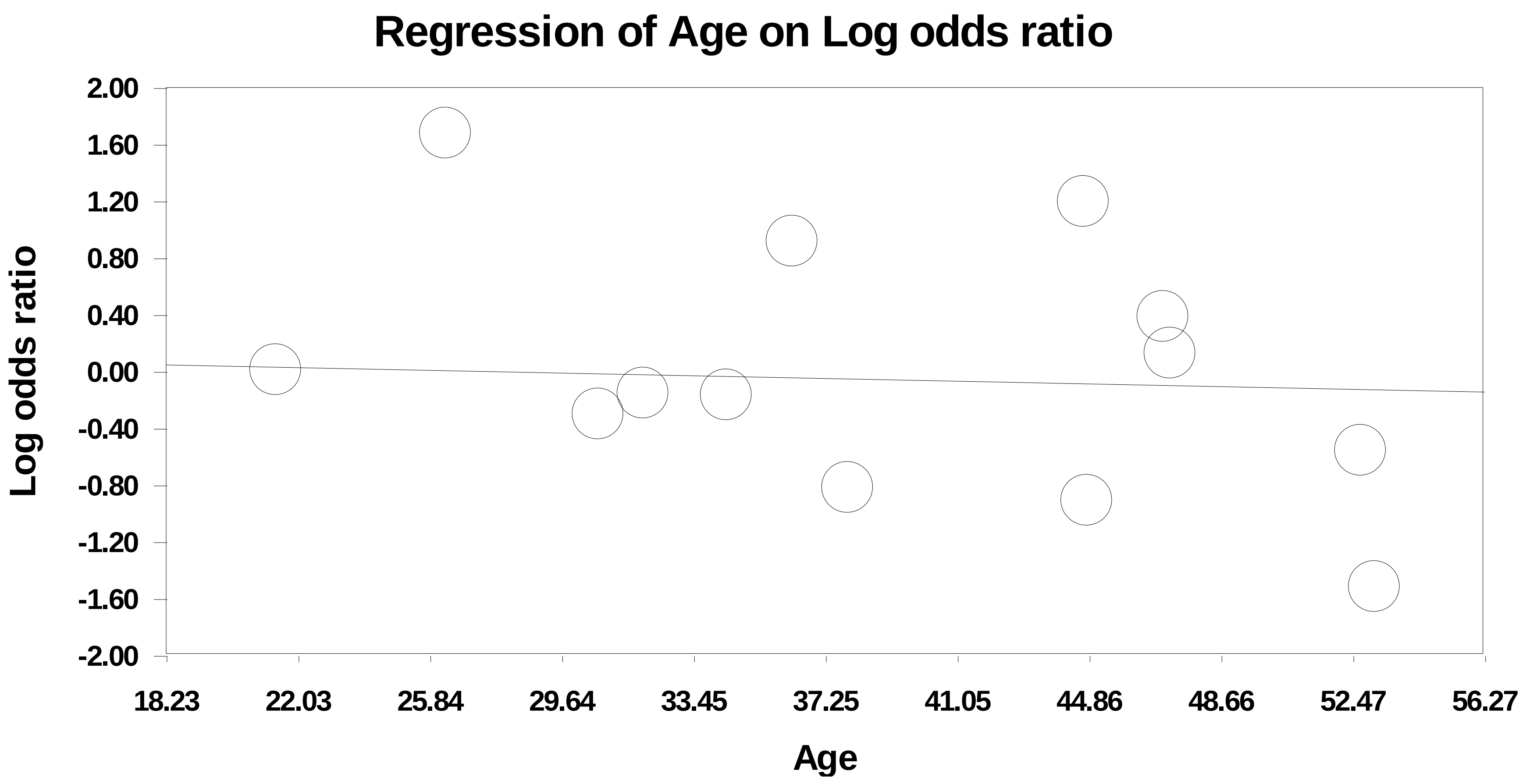

Supplement: Supplementary file 2 — Additional file 2. Association of the C957T polymorphism and schizophrenia. [file 12993_2016_114_MOESM2_ESM.pdf]
