# Supplementary material for: The role of C957T, TaqI and Ser311Cys polymorphisms of the DRD2 gene in schizophrenia: systematic review and meta-analysis
Source: Behav Brain Funct. 2016 Nov 9;12:29. doi: 10.1186/s12993-016-0114-z (PMC5101652; doi:10.1186/s12993-016-0114-z)
Supplement: Supplementary file 3 — Additional file 3. Sensitivity analysis and cumulative meta-analysis. [file 12993_2016_114_MOESM3_ESM.pdf]

Figure S1. Sensitivity analysis of C957T polymorphism with SZ in whole population: a) Allelic b) Additive c) Dominant and d) Recessive models.

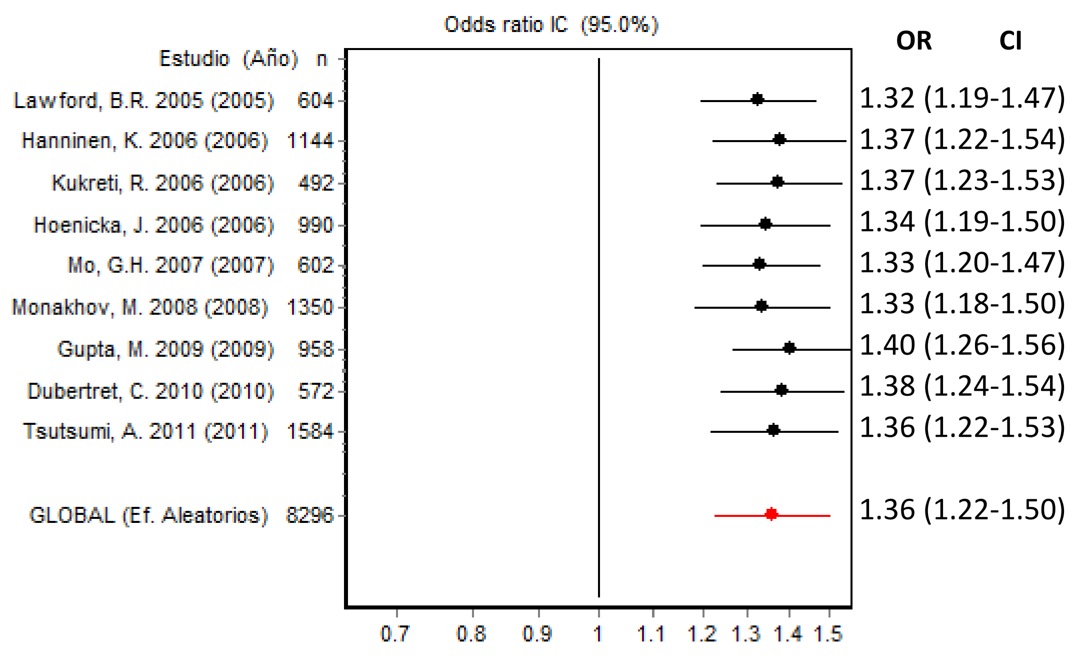

A)

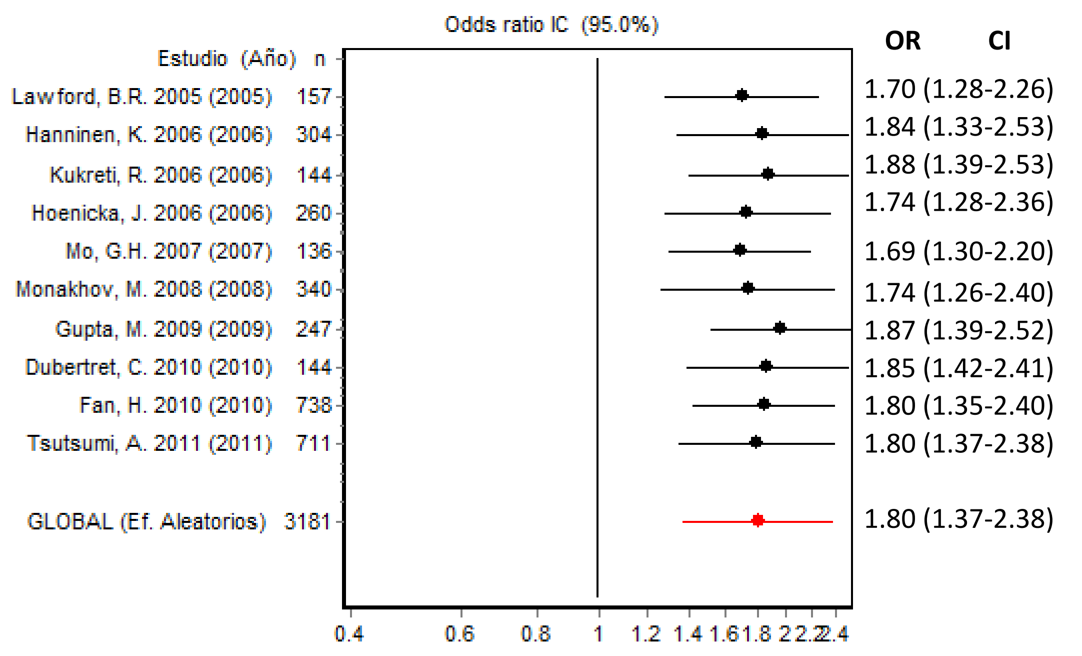

B)

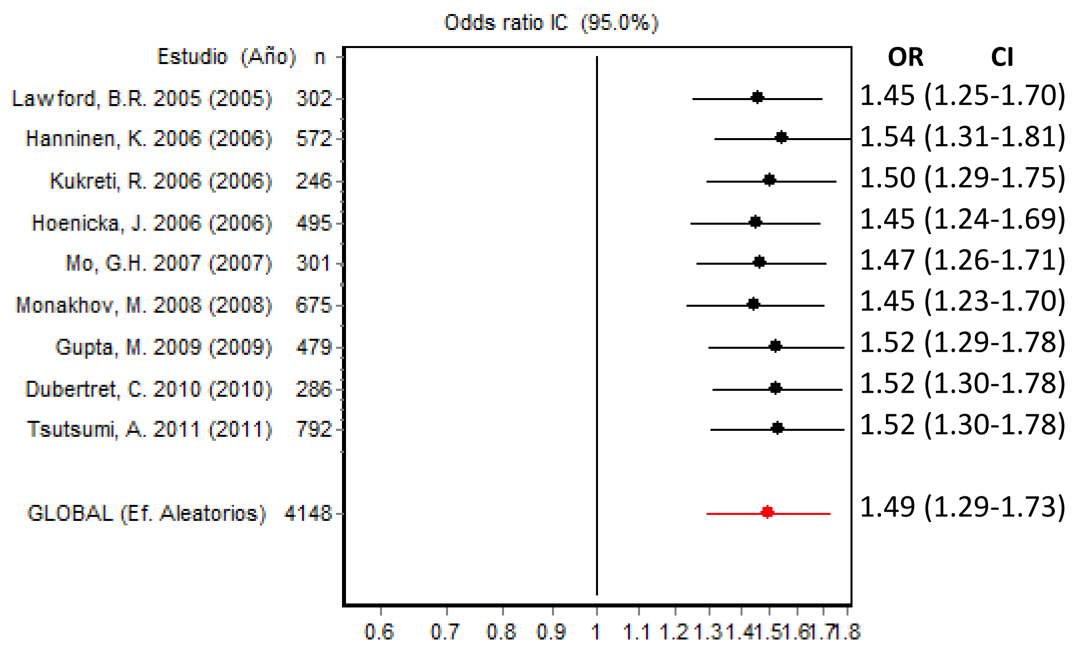

C)

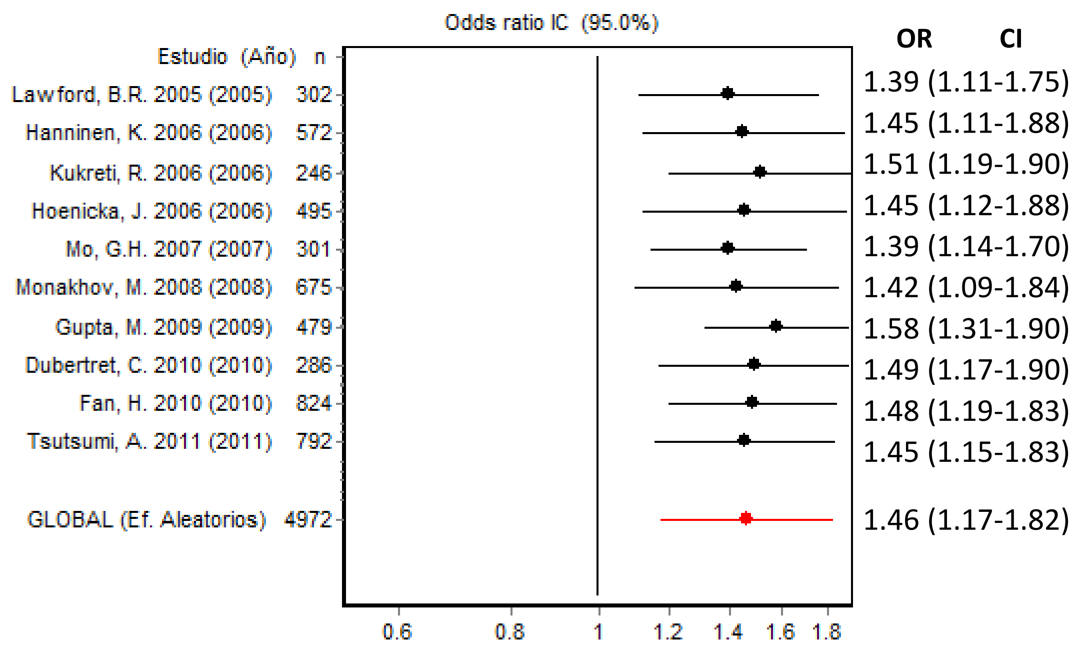

D)

Figure S2. Sensitivity analysis of C957T polymorphism with SZ in Caucasians population: a) Allelic b) Additive c) Dominant and d) Recessive models.

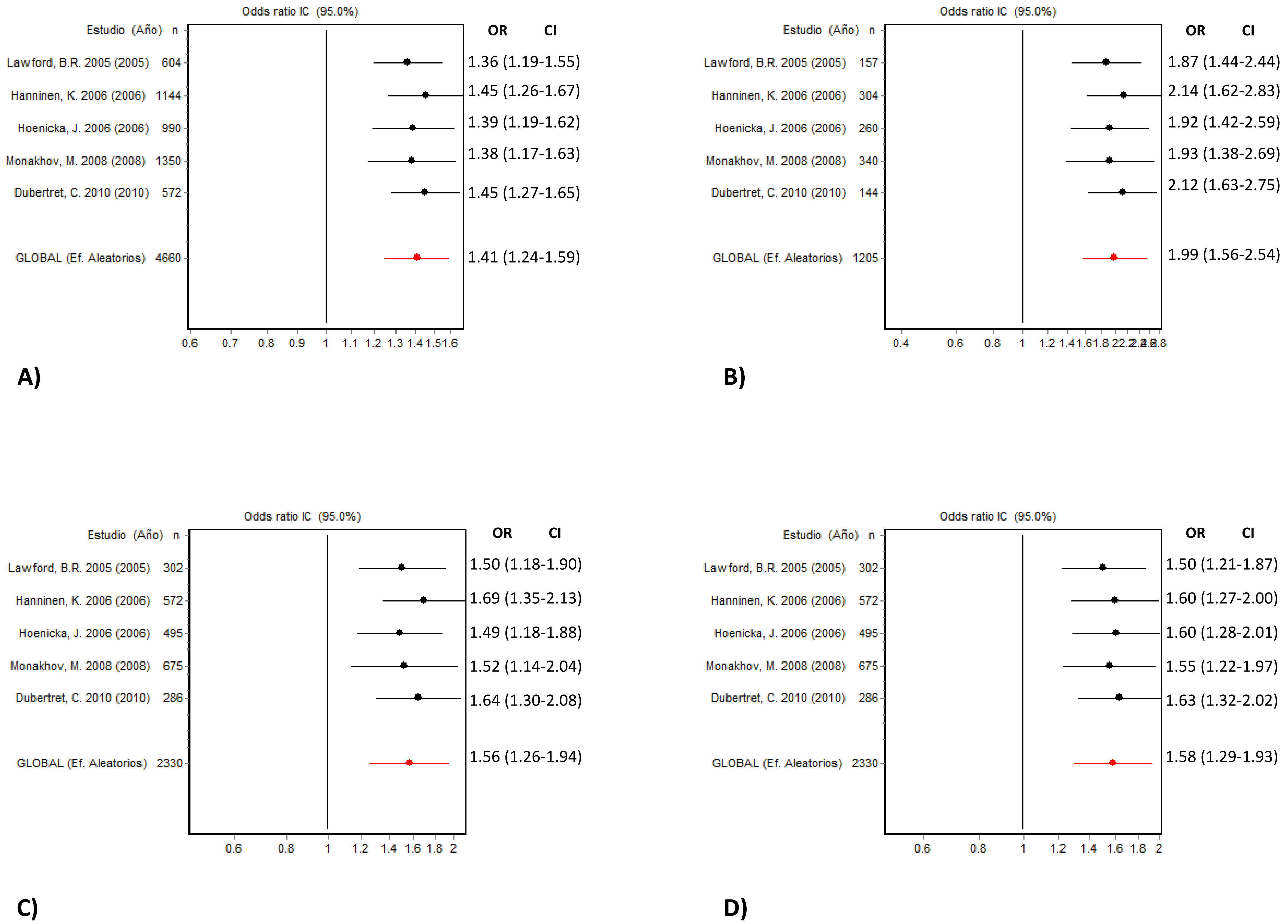

Figure S3. Sensitivity analysis of C957T polymorphism with SZ in Asians population: a) Allelic b) Additive c) Dominant and d) Recessive models.

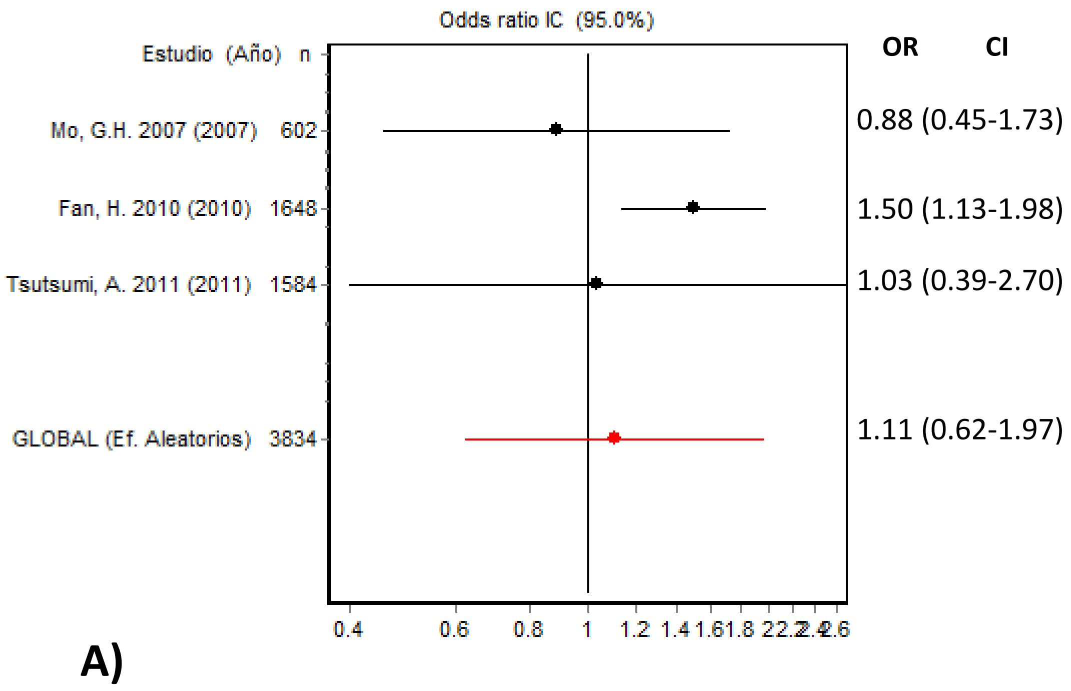

A)

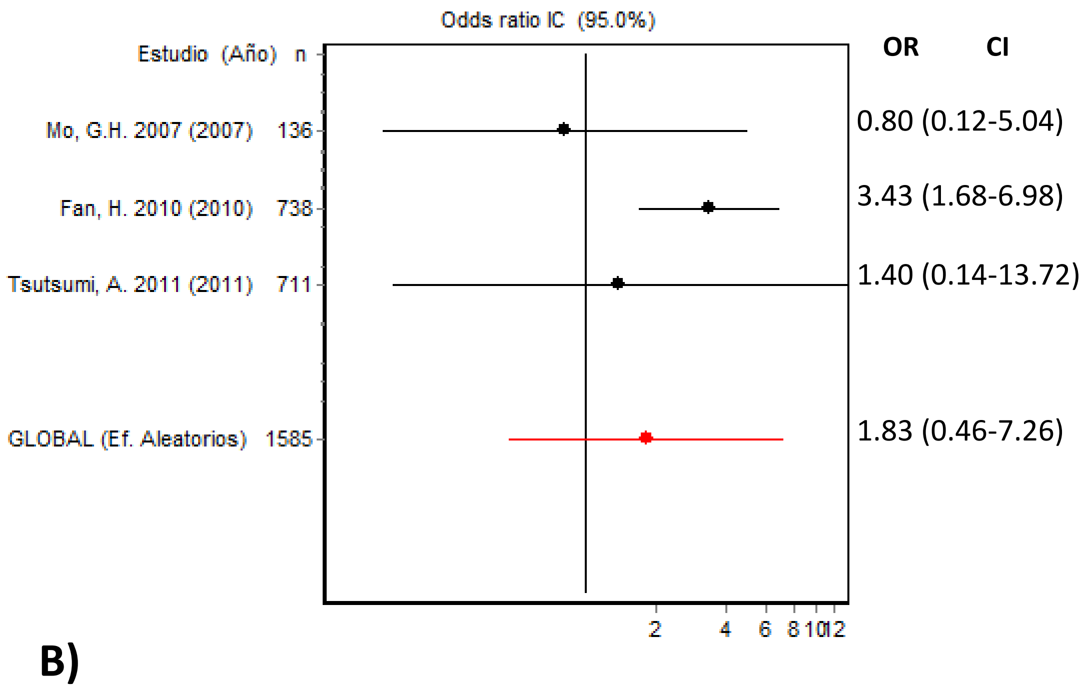

B)

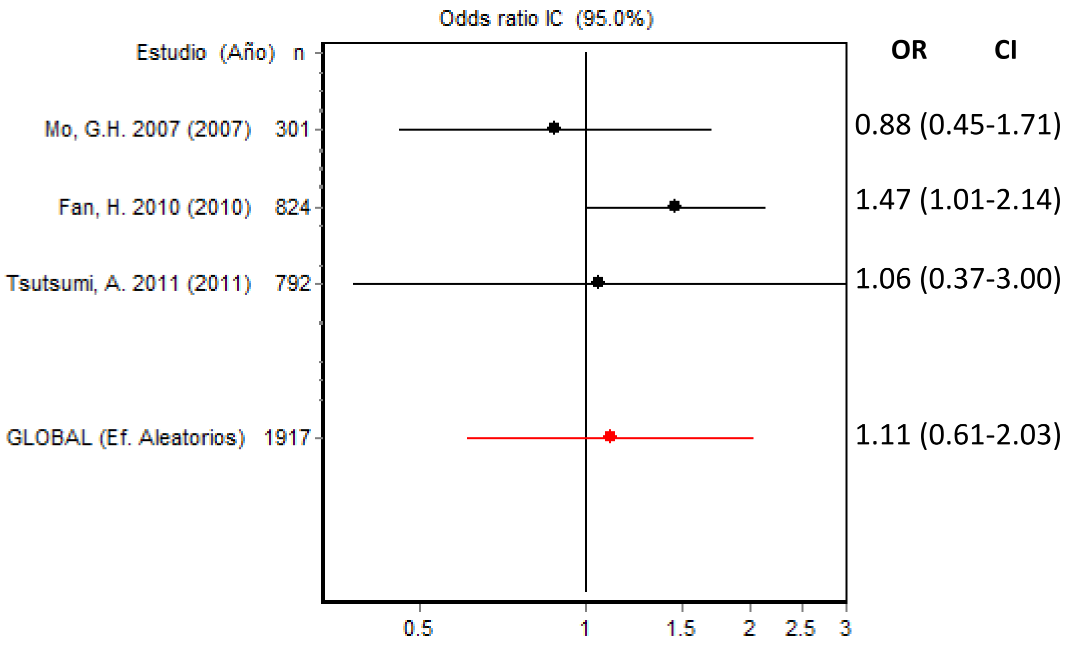

C)

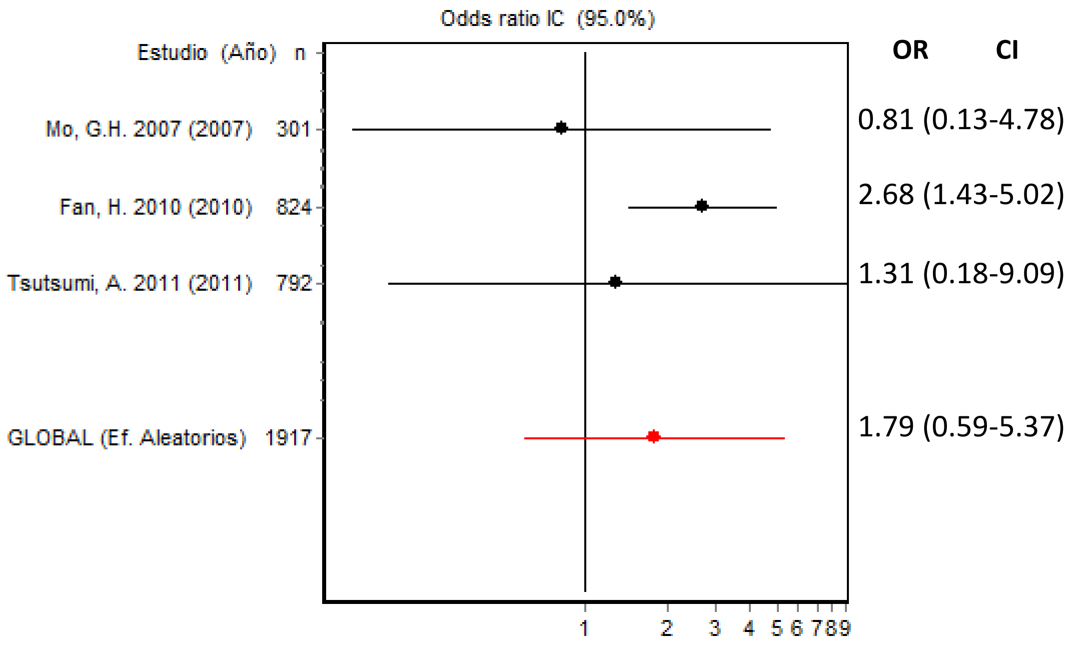

D)

Figure S5. Sensitivity analysis of TaqI polymorphism with SZ in whole population: a) Allelic b) Additive c) Dominant and d) Recessive models.

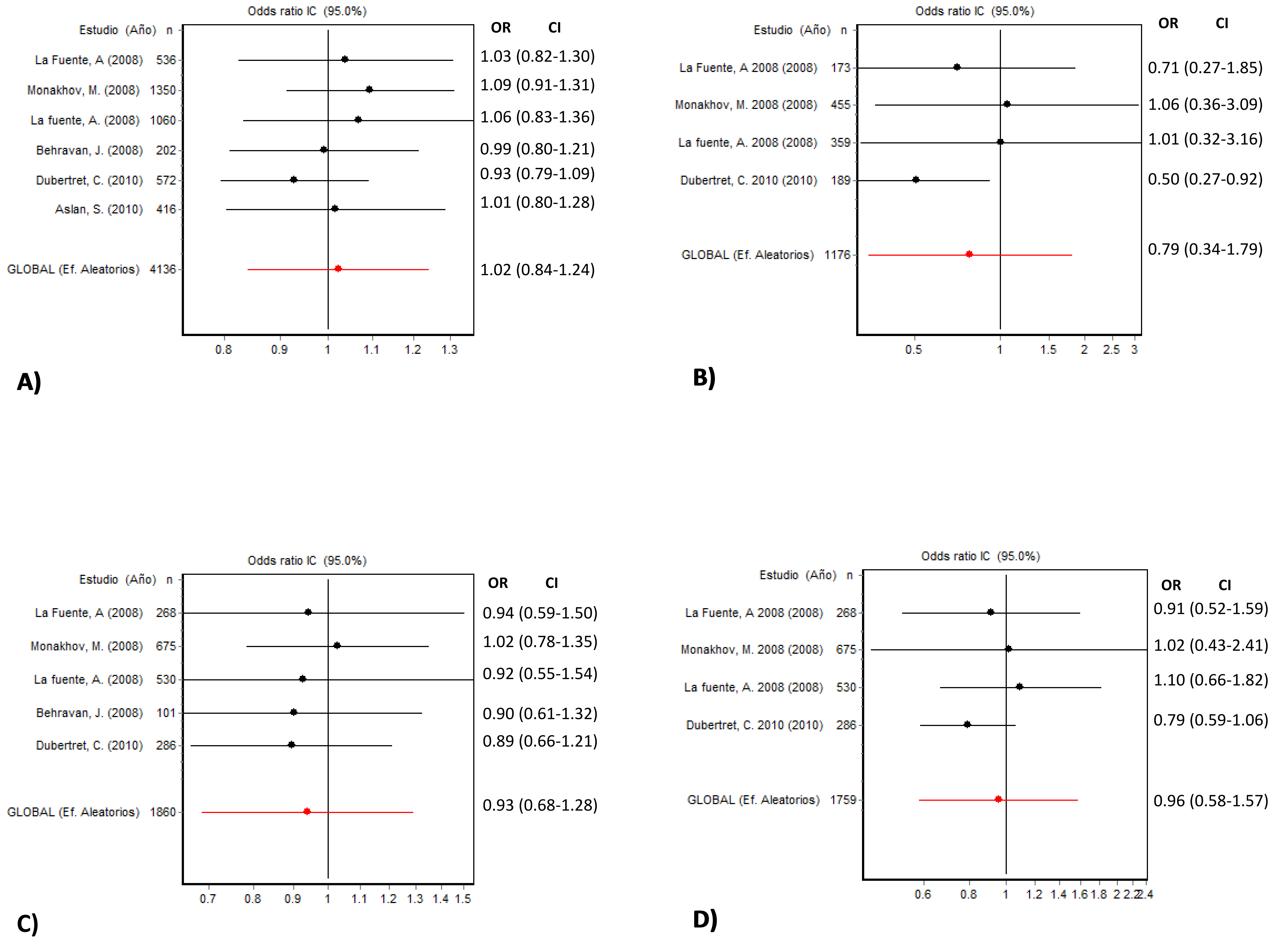

Figure S6. Sensitivity analysis of TaqI polymorphism with SZ in Caucasians population: a) Allelic b) Additive c) Dominant and d) Recessive models.

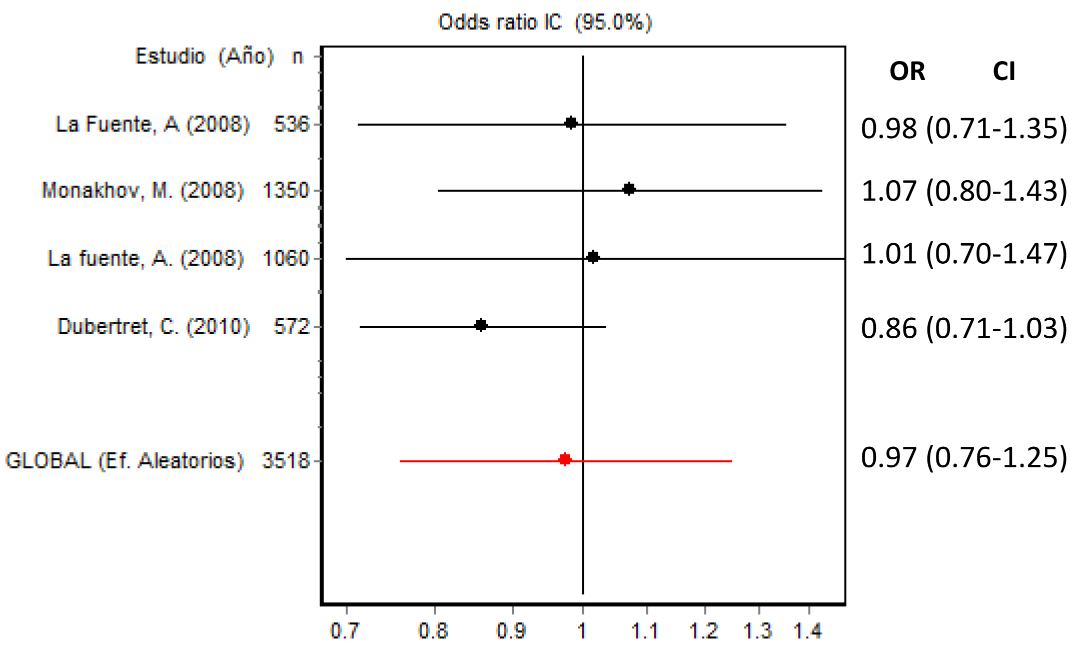

A)

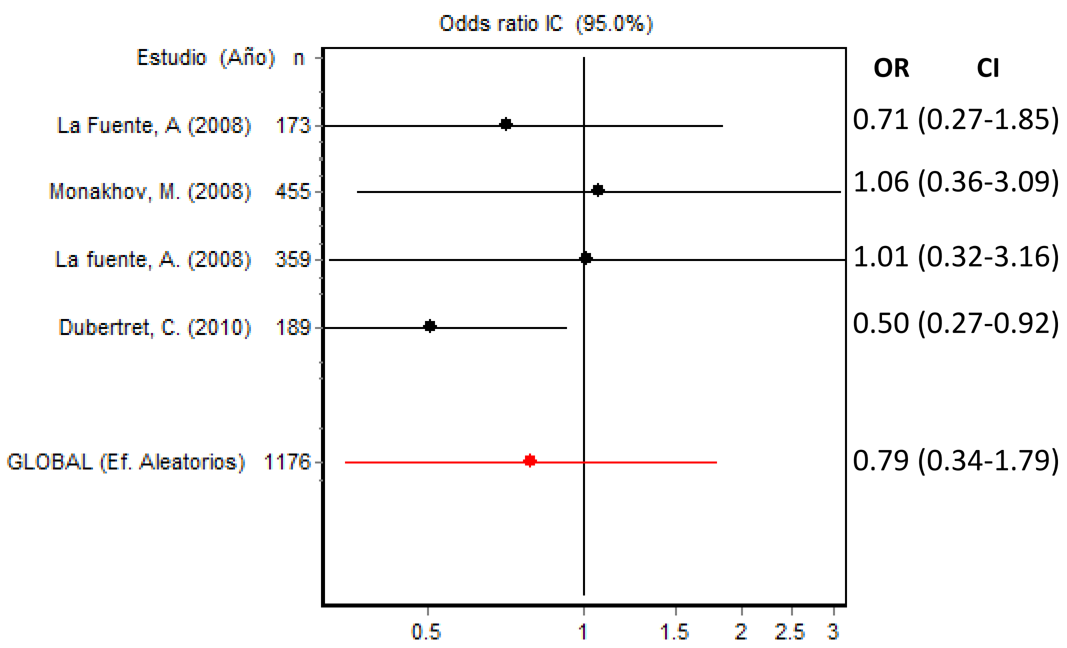

B)

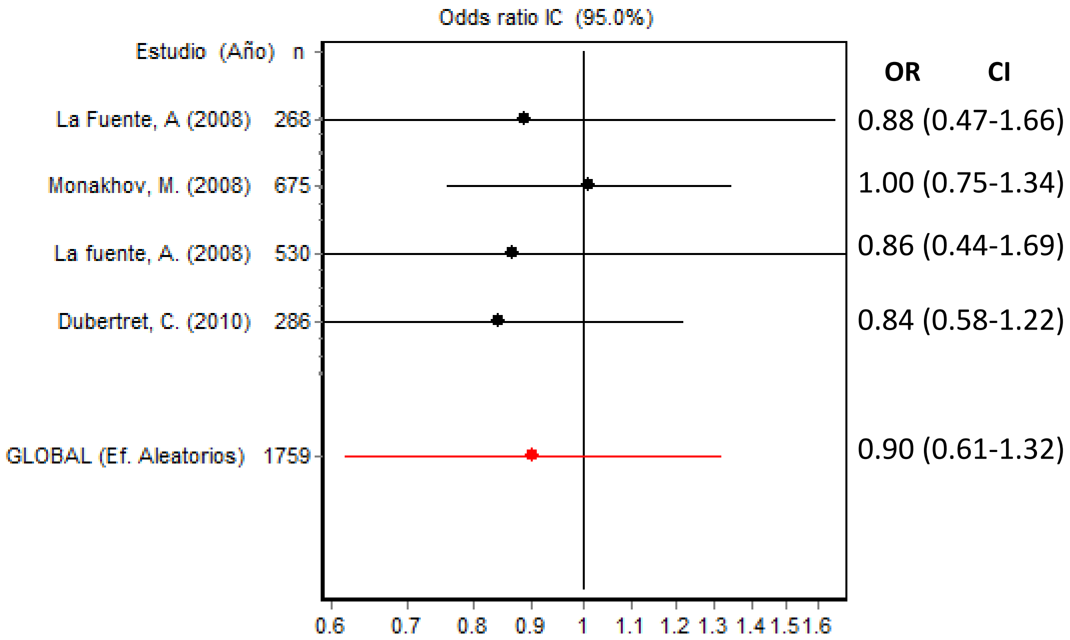

C)

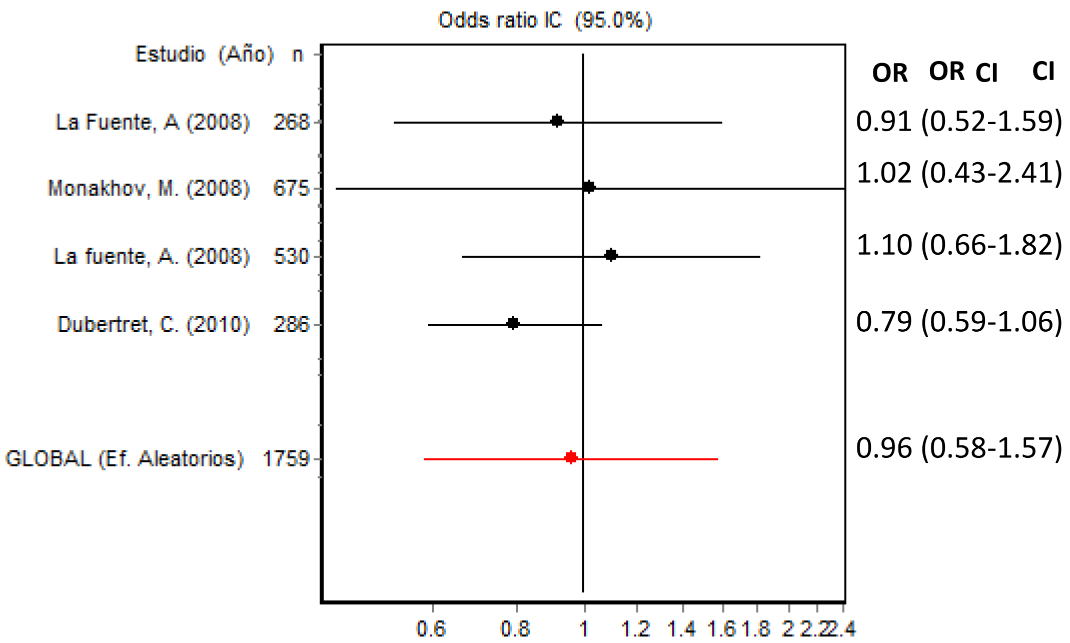

D)
